# SKP2 drives the sensitivity to neddylation inhibitors and cisplatin in malignant pleural mesothelioma

**DOI:** 10.1186/s13046-022-02284-7

**Published:** 2022-02-23

**Authors:** Iris Chiara Salaroglio, Dimas Carolina Belisario, Paolo Bironzo, Preeta Ananthanarayanan, Luisa Ricci, Sabrina Digiovanni, Simona Fontana, Francesca Napoli, Alberto Sandri, Chiara Facolmatà, Roberta Libener, Valentina Comunanza, Federica Grosso, Elena Gazzano, Francesco Leo, Riccardo Taulli, Federico Bussolino, Luisella Righi, Mauro Giulio Papotti, Silvia Novello, Giorgio Vittorio Scagliotti, Chiara Riganti, Joanna Kopecka

**Affiliations:** 1 Department of Oncology, University of Torino, via Santena 5/bis, 10126 Torino, Italy; 2 Thoracic Unit and Medical Oncology Division, Department of Oncology at San Luigi Hospital, University of Torino, Orbassano, Italy; 3 Present address: IRCCS San Raffaele Hospital DIBIT, 20132 Milano, Italy; 4 Pathology Unit, San Luigi Hospital, University of Torino, Orbassano, Italy; 5 Candiolo Cancer Institute, FPO – IRCCS, Candiolo, Italy; 6 Present address: German Cancer Research Center (DKFZ) and Technical University Munich, 81675 Munich, Germany; 7 Department of Integrated Activities Research and Innovation, S. Antonio and Biagio Hospital, Alessandria, Italy; 8 Oncology Division, S. Antonio and Biagio Hospital, Alessandria, Italy; 9 Interdepartmental Research Center of Molecular Biotechnology, University of Torino, Torino, Italy; 10 Present address: Department of Life Sciences and Systems Biology, University of Torino, 10123 Torino, Italy; 11 Thoracic Surgery Division, San Luigi Hospital, University of Torino, Orbassano, Italy; 12 Pathology Unit, City of Health and Science University Hospital, Torino, Italy

**Keywords:** Malignant pleural mesothelioma, SKP/Cullin/F-box complex, endoplasmic reticulum stress, immunogenic cell death, Pevonedistat

## Abstract

**Background:**

The combination of pemetrexed and cisplatin remains the reference first-line systemic therapy for malignant pleural mesothelioma (MPM). Its activity is moderate because of tumor aggressiveness, immune-suppressive environment and resistance to chemotherapy-induced immunogenic cell death (ICD). Preliminary and limited findings suggest that MPM cells have deregulated ubiquitination and proteasome activities, although proteasome inhibitors achieved disappointing clinical results.

**Methods:**

Here, we investigated the role of the E3-ubiquitin ligase SKP/Cullin/F-box (SCF) complex in cell cycle progression, endoplasmic reticulum (ER)/proteostatic stress and ICD in MPM, and the therapeutic potential of the neddylation/SCF complex inhibitor MLN4924/Pevonedistat.

**Results:**

In patient-derived MPM cultures and syngenic murine models, MLN4924 and cisplatin showed anti-tumor effects, regardless of MPM histotype and BAP1 mutational status, increasing DNA damage, inducing S- and G2/M-cell cycle arrest, and apoptosis. Mechanistically, by interfering with the neddylation of cullin-1 and ubiquitin-conjugating enzyme UBE2M, MLN4924 blocks the SCF complex activity and triggers an ER stress-dependent ICD, which activated anti-MPM CD8^+^T-lymphocytes. The SKP2 component of SCF complex was identified as the main driver of sensitivity to MLN4924 and resistance to cisplatin. These findings were confirmed in a retrospective MPM patient series, where SKP2 high levels were associated with a worse response to platinum-based therapy and inferior survival.

**Conclusions:**

We suggest that the combination of neddylation inhibitors and cisplatin could be worth of further investigation in the clinical setting for MPM unresponsive to cisplatin. We also propose SKP2 as a new stratification marker to determine the sensitivity to cisplatin and drugs interfering with ubiquitination/proteasome systems in MPM.

**Supplementary Information:**

The online version contains supplementary material available at 10.1186/s13046-022-02284-7.

## Background

Malignant pleural mesothelioma (MPM) is an aggressive type of cancer primarily caused by asbestos exposure, characterized by a long latency period (up to 50 years) and an expected increased incidence in the next decades [1]. Histologically, MPM is classified in three subtypes: epithelioid, sarcomatous and biphasic, representing 60%, 20% and 10% of MPMs at the diagnosis, respectively. Since MPM is usually diagnosed at late stage, chemotherapy is often the only therapeutic option considered [2]. The first-line standard regimen consists of cisplatin and pemetrexed, which confers a median overall survival (OS) of 12 months only and a median progression free survival (PFS) of less than 6 months. The poor intrapleural drug delivery, the increased drug efflux via plasma-membrane transporters [3], the presence of chemo-refractory stem cells [4], the highly immune-suppressive environment [5, 6] partially explain the low success rate of chemotherapy. MPM cells are refractory to immunogenic cell death (ICD) [3], i.e. an immune system-dependent cell death that occurs after endoplasmic reticulum (ER) stress or DNA damage elicited by chemotherapy [7]. This is characterized by the exposure of calreticulin (CRT), the release of ATP and high mobility group protein (HMGB1), followed by phagocytosis of tumor cells by dendritic cells (DCs) and activation of cytotoxic CD8^+^ T-lymphocytes [7]. Cisplatin triggers ER stress [8], damages DNA and hinders DNA repair [9], but these events are not sufficient to elicit ICD in MPM [3].

Previously, we reported that MPM has deregulation of ubiquitination and proteasomal protein degradation [3, 10] and that the proteasome inhibitor carfilzomib restored ICD in cisplatin-treated cells [3]. However, in two clinical trials (NCT00513877 and NCT00458913) bortezomib did not improve the survival of MPM patients, because of the high inter-patient variability in the expression of 20S proteasome subunits that modify bortezomib sensitivity [11].

With the aim of identifying alternative inducers of proteostatic and ICD effective in MPM, we focused on neddylation inhibitors [12, 13], particularly on MLN4924/Pevonedistat. MLN4924 inhibits the neural precursor cell expressed developmentally down-regulated 8 (NEDD8) activating enzyme [14, 15], which activates cullin proteins in the E3-ubiquitin ligase S-Phase Kinase Associated Protein SKP/Cullin/F-box (SCF) complex [16]. Interestingly, 10% MPM patients have missense mutations in the *Cullin 1* gene [17], suggesting that SCF complex may play a role in MPM progression.

We demonstrated a strong synergism between cisplatin and MLN4924 in patient-derived cisplatin-resistant MPM cells, based on the induction of DNA damage and ER stress that triggers ICD. We identified SKP2 as a stratification factor that determines the response of MPM to cisplatin and MLN4924.

## Methods

### Chemicals

Plasticware was from Falcon (Becton Dickinson, Franklin Lakes, NJ). MLN4924 was obtained from Cayman Chemical (Ann Arbor, MI). If not specified otherwise, reagents were obtained from Sigma Chemicals Co. (St. Louis, MO).

### Cells

Twenty-three primary human MPM cultures (13 epithelioid, 5 biphasic, 5 sarcomatous), obtained during diagnostic thoracoscopies, were collected from the San Luigi Gonzaga Hospital (Orbassano, Italy), the Città della Salute e della Scienza Hospital (Torino, Italy) and the Biologic Bank of Malignant Mesothelioma, S. Antonio e Biagio e Cesare Arrigo Hospital (Alessandria, Italy), after written informed consent. The local Ethical Committees approved the study (#9/11/2011; #126/2016). Cultures were used within passage 10. Clinical and pathological characteristics of the MPM patients are reported in the Additional Table 1, histopathological features of MPM [5] in the Additional Table 2. Murine AB1 cells were obtained from Sigma Chemicals Co. (#10092305). Primary MPM cells and AB1 cells were grown, respectively, in HAM’s F12 and DMEM medium, supplemented with 10% v/v fetal bovine serum and 100 U/ml penicillin-100 μg/ml streptomycin.

### Immunoblotting

Cells were rinsed with lysis buffer (50 mM Tris-HCl, 1 mM EDTA, 1 mM EGTA, 150 mM NaCl, 1% v/v Triton-X100; pH 7.4), supplemented with the protease inhibitor cocktail set III, 2 mM phenylmethanesulfonyl fluoride and 1 mM Na_3_VO_4_, sonicated and centrifuged (13,000 × g, for 10 min at 4°C). 20 μg proteins were probed with the following antibodies: cullin 1 (12895-1-AP, Proteintech Group, Inc., Chicago, IL), recognizing both neddylated and unneddylated cullin 1; SKP1 (sc-5281, Santa Cruz Biotechnology Inc.); SKP2 (15010-1-AP, Proteintech Group Inc.); Heat Shock 70kDa Protein 5/Binding Immunoglobulin Protein/Glucose-Regulated Protein 78kDa (HSP5A/BiP/GRP78; NBP2-16749, Novus Biological, Centennial, CO); Eukaryotic Translation Initiation Factor 2 α Kinase 3/protein kinase R-like endoplasmic reticulum kinase (EIF2AK3/PERK; sc-377400, Santa Cruz Biotechnology Inc.); β-tubulin (sc-52-74, Santa Cruz Biotechnology Inc.), followed by peroxidase-conjugated secondary antibodies (Bio-Rad Laboratories, Hercules, CA). Blots were washed with Tris-buffered saline/Tween 0.01% v/v, developed with enhanced chemiluminescence (Bio-Rad Laboratories) and imaged using a ChemiDoc^TM^ Touch Imaging System device (Bio-Rad Laboratories).

### Cell cytotoxicity, viability and proliferation

Lactate dehydrogenase (LDH) release, considered an index of cell necrosis, was measured spectrophotometrically as reported in [4]. Cristal violet staining was used to assess cell viability [10], using a Synergy HT 96-well microplate reader (Bio-Tek Instruments, Winooski, VT). The mean absorbance of untreated cells was considered 100%; the absorbance units were expressed as percentage of viable cells vs. untreated cells. Cells proliferation were measured using 5 [6]-carboxyfluorescein diacetate N-succinimidyl ester (CFSE) staining. Briefly, 1×10^6^ cells were detached and stained for 8 min with 5μM CFSE in PBS, then washed, seeded in 6 well plates and incubated with the drugs as indicated in the Results section. After 24h and 48h the cells were detached and the intracellular fluorescence was measured using a Synergy HT 96-well microplate reader, using a 480 nm and 520 nm as excitation and emission wavelength. The mean fluorescence of untreated cells was considered 1; the relative fluorescence units were expressed as fold change of treated vs. untreated cells. The Combination Index (CI) was calculated using the CalcuSyn software (www.biosoft.com/w/calcusyn.html), using the Chou-Talalay algorithm [18].

### *In vivo* tumor growth

6-week-old female immunocompetent BALB/C mice (Charles River Laboratories Italia, Calco) were housed (5 per cage) under 12 h light/dark cycle, with food and drinking provided ad libitum. Mice were subcutaneously (s.c.) inoculated with 1×10^6^ AB1 cells mixed with 100 μl Matrigel. Tumor growth was measured daily by caliper, according to the equation (LxW^2^)/2, where L=tumor length and W=tumor width. When the tumor volume reached 50 mm^3^, animals were randomized into 4 groups (n = 10 animals/group) and treated for 3 consecutive weeks as follows: 1) control group, treated with 0.1 ml saline solution intraperitoneally (i.p.) once a week; 2) cisplatin group, treated with 5 mg/kg cisplatin i.p. once a week; 3) MLN4924 group, treated with 25 mg/kg MLN4924 s.c. 5 days/week; 4) cisplatin plus MLN4924 group, treated with both drugs as above. Tumor volumes were monitored and animals were euthanized 21 days after randomization. Blood was collected immediately after euthanasia and used for hemato-chemical analyses using the respective kits from Beckman Coulter Inc. (Beckman Coulter, Miami, FL). The experimental procedures were approved by the Bio-Ethical Committee of the Italian Ministry of Health (#122/2015-PR).

### Immune-infiltrate and immunohistochemistry analysis

After resection, tumors were digested to obtain a single cell suspension. Tumor infiltrating lymphocytes (TILs) were isolated from 2×10^6^ single-cell suspension derived from the homogenated tumors, using the CD8 (TIL) MicroBeads (mouse, 130-116-478, Miltenyi Biotec., Bergisch Gladbach, Germany) and the CD4 (TIL) MicroBeads (mouse, 130-116-475, Miltenyi Biotec.), in order to obtained a purified population of TILs that represent a small percentage of cells contained in the tumor mass, ready for the quantification by flow cytometry. TIL quantification was performed using a Guava EasyCyte flow cytometer (Millipore, Bedford, MA), equipped with the InCyte software (Millipore). For immunohistochemical analyses, tumors sections were stained for Ki67 (AB9260, Millipore), CD4 (ab183685, Abcam, Cambridge, UK), CD8 (ab22378, Abcam) antibodies, followed by a secondary HRP-labelled antibody (Dako, Glostrup, Denmark). For the detection of intratumor apoptosis, tumor sections were treated with the In Situ Cell Death Detection Kit (11684795910, Sigma Chemicals Co.) based on TUNEL staining. Sections were examined with a Leica DC100 microscope (Leica Microsystems GmbH Wetzlar, Germany). Immunohistochemical imaging quantification was performed with ImageJ software (https://imagej.nih.gov/).

### Cell cycle analysis, senescence, autophagy, DNA damage and apoptosis

For the cell cycle analysis, 1×10^5^ cells were fixed in 70%v/v ethanol for 15 min, then centrifuged at 13000 rpm for 5 min at 4°C rinsed with citrate buffer (50 mM Na_2_HPO_4_, 25 mM sodium citrate, 1% v/v Triton X-100), containing 1 μg/ml propidium iodide and 1 μg/ml RNAse, and analysed after 15 min incubation in the dark. For each flow cytometry analysis, 20000 events were collected using Guava®easyCyte flow cytometer (Millipore, Billerica, MA, USA). Cell cycle distribution was analysed using InCyte software (Millipore) [5]. Senescence was detected using the Senescence Cells Histochemical Staining Kit (CS0030, Sigma Chemicals Co.) based on the β-galactosidase staining. Autophagy was measured with the Autophagy Assay Kit (ab139484, Abcam). DNA damage and apoptosis were measured with the DNA Fragmentation Imaging Kit (06432344001, Roche, Mannheim, Germany).

### Tumor Mutational Burden (TMB)

DNA extraction was performed using GenElute Mammalian Genomic DNA Purification Kit (G1N70, Sigma Chemicals Co.). Whole exome library was prepared using SureSelect v7 (Agilent Technologies, Santa Clara, CA), sequenced using the Illumina NovaSeq 6000 S4 2x150bp Flow Cell. TMB (number of exonic variants per Mb) was calculated using the SureSelect Human All Exon V7 software (Agilent Technologies). Sequencing and bioinformatics analysis were performed by Biodiversa srl (Rovereto, Italy).

### Protein ubiquitination assay, proteasome activity

Ubiquitination was measured with the E3Lite Customizable Ubiquitin Ligase kit (UC101, Life-Sensors Inc., Malvern, PA), using 5 nM E1 activating enzyme, 100 nM E2 conjugating enzyme UBE2M, 200 μM ATP, 6 mM human recombinant ubiquitin. Proteasome activity was measured with the Proteasome-Glo™ Cell-Based Assays (PRG8860, Promega Corporation, Madison, WI).

### Calreticulin exposure, ATP and HMGB1 release

Surface calreticulin was measured as reported in [3], using Guava® easyCyte flow cytometer (Millipore). Control samples were incubated with non-immune isotypic antibody. 100 μl of culture medium were used to measure ATP with the ATP Bioluminescent Assay Kit (FL-AA, Sigma Chemicals Co.) and HMGB1 with the Enzyme-linked Immunosorbent Assay Kit for High Mobility Group Protein 1 (SEA399Hu, Cloud-Clone Corp., Katy, TX).

### Phagocytosis and T-lymphocyte activation

Blood Bank of AOU Città della Salute e della Scienza, Torino, Italy, provided healthy donors’ peripheral blood monocytes (#DG-767/2015) to generate DCs [3]. The phagocytosis rate of MPM cells, expressed as phagocytic index, was measured by flow cytometry [3]. T-lymphocytes were isolated by immuno-magnetic sorting with the Pan T Cell Isolation Kit (Miltenyi Biotec.) and co-cultured with DCs for 10 days at 1:5 ratio. The percentage of CD8^+^CD107a^+^T-lymphocytes, indicative of an active anti-tumor cytotoxic response, was determined by flow cytometry [3].

### *Cul1* mutational status

Primers for Sanger sequencing were designed to detect the hot spot codon 471 of the *Cul1* gene. Sequences of forward and reverse primers are: 5’-ATCTTGGCTCCTGGCTTGT-3’; 5’-CTCGGCCATAGAGCTGTTTT-3. PCR products (294 bp) were sequenced using an ABI PRISM 3730 (Applied Biosystems, Foster City, CA, USA). Sequences were confirmed twice, starting from independent PCR reactions.

### Real Time PCR (RT-PCR) and PCR array

Total RNA was extracted and reverse-transcribed using the iScript^TM^ cDNA Synthesis Kit (Bio-Rad Laboratories). qRT-PCR was carried out using IQ^TM^ SYBR Green Supermix (Bio-Rad Laboratories). qPrimerDepot software (http://primerdepot.nci.nih.gov/) was used to obtain the following PCR primers: *cullin 1*: 5’- GGATGTCCTTTAAATGTAGAGAATGA -3’, 5’- GAGGGACGCAGCTAGACCTT -3’; *SKP1*: 5’- CTCCTTCATCATCCATTCCC3’, 5’-CCGTCTCCTTAACACCGAAC-3’; *SKP2*: 5’- GGAAGGGAGTCCCATGAAA-3’, 5’-GCTGAAGAGCAAAGGGAGTG-3’; *S14*: 5’-CGAGGCTGATGACCTGTTCT-3’, 5’-GCCCTCTCCCACTCTCTCTT-3’. Gene Expression Quantitation software (Bio-Rad Laboratories) was used to assess relative gene expression levels. The PCR arrays were performed on 0.5 μg cDNA, using the Unfolded Protein Response (UPR) PCR Array (Bio-Rad Laboratories).

### SKP2 knockout/overexpression

2×10^5^ cells were transfected with 1 μg of: control CRISPR/Cas9 plasmid (sc-418922), Skp2 p45 CRISPR/Cas9 KO Plasmid (sc-400534-KO-2); control CRISPR Activation Plasmid (sc-437275), SKP2 CRISPR Activation Plasmid (sc-400534-ACT), all from Santa Cruz Biotechnology Inc., according to the manufacturer’s instructions. The efficacy of knockout or overexpression was controlled by RT-PCR and immunoblotting.

### Statistical analysis

Results were analyzed by a one-way analysis of variance (ANOVA) and Tukey’s test, using GraphPad Prism software (v 6.01); p values <0.05 were considered significant. The Kaplan-Meier method was used to estimate time to progression (TTP: time from the start of chemotherapy to the evidence of the disease progression) and OS (survival from the start of chemotherapy until patients’ death). Log rank test was used to compare the outcome of SKP2^*low*^ and SKP2^*high*^ groups. The sample size was calculated with the G*Power software (www.gpower.hhu.de), setting α<0.05 and 1-β=0.80. Laboratory researchers were unaware of the efficacy outcomes of the treatment received by the MPM patients.

## Results

### MLN4924 and cisplatin have a synergic anti-tumor effect in patient-derived mesothelioma cells and murine models

**Fig. 1:**
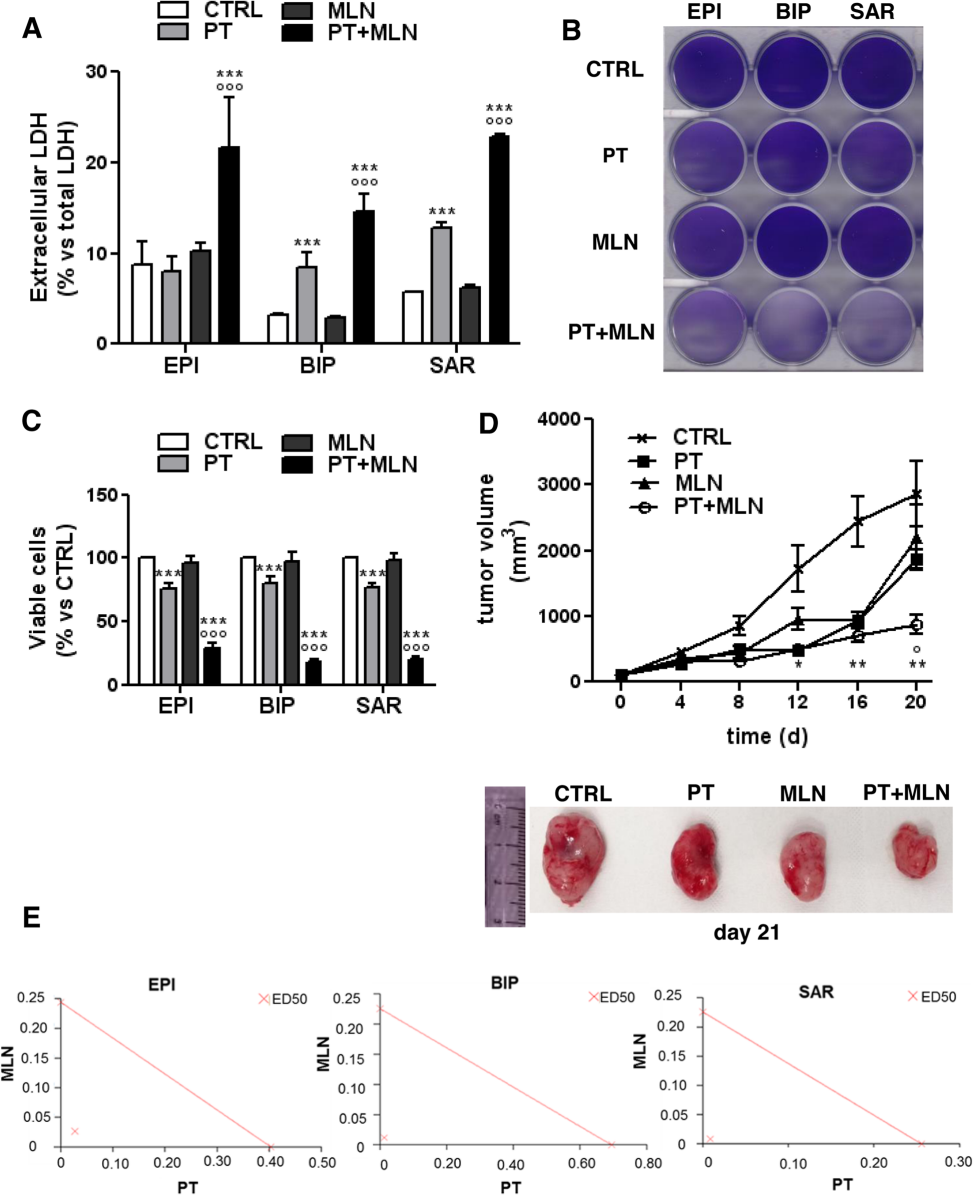
Cisplatin and MLN4924 combination induce mesothelioma cell death. Primary MPM cells derived from 3 different histopathological subtypes, epithelioid (EPI BAP1^+^ UPN7 and EPI BAP1^-^ UPN11), biphasic (BIP BAP1^+^ UPN14 and BIP BAP1^-^ UPN16) and sarcomatous (SAR BAP1^+^ UPN22 and SAR BAP1^-^ UPN23), were incubated 48 h in fresh medium (CTRL), with 50 μM cisplatin (PT), 0.2 μM MLN4924 (MLN) or their combination (PT+MLN). **A**. LDH release was measured spectrophotometrically in triplicates. Data are presented as means of 2 primary MPM for each histopathological subtype used + SD (*n* = 3). ***p < 0.001: treated cells vs CTRL cells; °°°*p* < 0.001: PT+MLN-treated cells vs PT-treated cells. **B**. Representative crystal violet staining of epithelioid (EPI; UPN7), biphasic (BIP; UPN16) and sarcomatous (SAR; UPN22) MPM cells. The photographs are representative of 1 out of 3 experiments, performed in quadruplicates. **C**. Crystal violet quantification after incubation for 72 h in fresh medium (CTRL), with 50 μM cisplatin (PT), 0.2 μM MLN4924 (MLN) or their combination (PT+MLN) of epithelioid (EPI BAP1^+^ UPN7 and EPI BAP1^-^ UPN11), biphasic (BIP BAP1^+^ UPN14 and BIP BAP1^-^ UPN16) and sarcomatous (SAR BAP1^+^ UPN22 and SAR BAP^-^ UPN23) MPM cells. Data are presented as means of 2 primary MPM for each histopathological subtype used + SD (*n* = 3), performed in quadruplicates. ***p < 0.001: treated cells vs CTRL cells; °°°*p* < 0.001: PT+MLN-treated cells vs PT -treated cells. **D**. AB1 cells were subcutaneously implanted into 6-week-old female BALB/c mice, when the tumor size reached 50 mm^3^ mice (*n* = 10 animals/group) were treated for 3 consecutive weeks as indicated in the Methods section. *Upper panel:* Tumor growth was monitored twice a week by caliber measurement. Data are presented as means ± SD. *p < 0.05, **p < 0.01: all groups vs CTRL group; °*p* < 0.05: PT+MLN group vs PT group. *Lower panel:* Representative photographs of tumors from each group. **E**. Epithelioid (EPI BAP1^+^ UPN7), biphasic (BIP BAP1^+^ UPN14) and sarcomatous (SAR BAP1^+^ UPN22) cells, i.e. one MPM representative per each histotype, were incubated with scalar concentrations (0, 10^-10^, 10^-9^, 10^-8^, 10^-7^, 10^-6^, 10^-5^, 10^-4^, 10^-3^ M) of MLN4924 and cisplatin for 72 h, then the viability was assessed by the crystal violet staining. The Combination Index (CI) was calculated using the CalcuSyn software. Results are representative of 3 independent experiments, performed in quadruplicates. ED50: dose that effectively reduces cell viability at 50% with single agent or their combination

Initially we tested the anti-tumor efficacy of MLN4924, alone or combined with cisplatin, in 6 patient-derived MPM cultures, including all subtypes and BAP1^+^/BAP1^-^ combinations (Additional Table 1). Cisplatin was used at a concentration falling in the IC_25_-IC_75_ range already determined in these MPM samples [4] and MLN4924 at a concentration corresponding to the IC_50_ observed in most solid cancer cells [19]. While cisplatin exerted its effect in terms of increase of cell necrosis, measured by LDH release (Fig. 1A), decrease in cell viability, measured by crystal violet staining, (Fig. 1B-C) and decrease in cell proliferation, measured by CSFE staining (Additional Fig. 1), MLN4924 as single agent did not. Interestingly, the combination of cisplatin and MLN4924 was more active than cisplatin alone in increasing necrosis (Fig. 1A), decreasing cell viability, (Fig. 1B-C) cell proliferation (Additional Fig. 1), independently of the histotypes. Accordingly, AB1 murine mesotheliomas implanted in syngeneic BALB/c mice showed low sensitivity to cisplatin, as already observed with this MPM line [3] and MLN4924 alone, notwithstanding its proven efficacy as single agent against different solid tumors [14]. Conversely, the combination of cisplatin and MLN4924 was significantly more cytotoxic, as indicated by the lower tumor growth and the smaller size of tumors excised after the treatment (Fig. 1D). Based on the haemato-chemical parameters (Additional Table 3) none of the above treatments induced liver, kidney, and heart toxicity. Notably, the CI of cisplatin and MLN4924, measured in three MPM with different histotype, was 0.11098, 0.08012 and 0.07263 for epithelioid, biphasic and sarcomatous MPM (Fig. 1E), indicating a very strong synergistic effect of the two agents, independent from the MPM histotype.

### MLN4924 enhances the cell cycle arrest, DNA damage and cellular senescence elicited by cisplatin

**Fig. 2:**
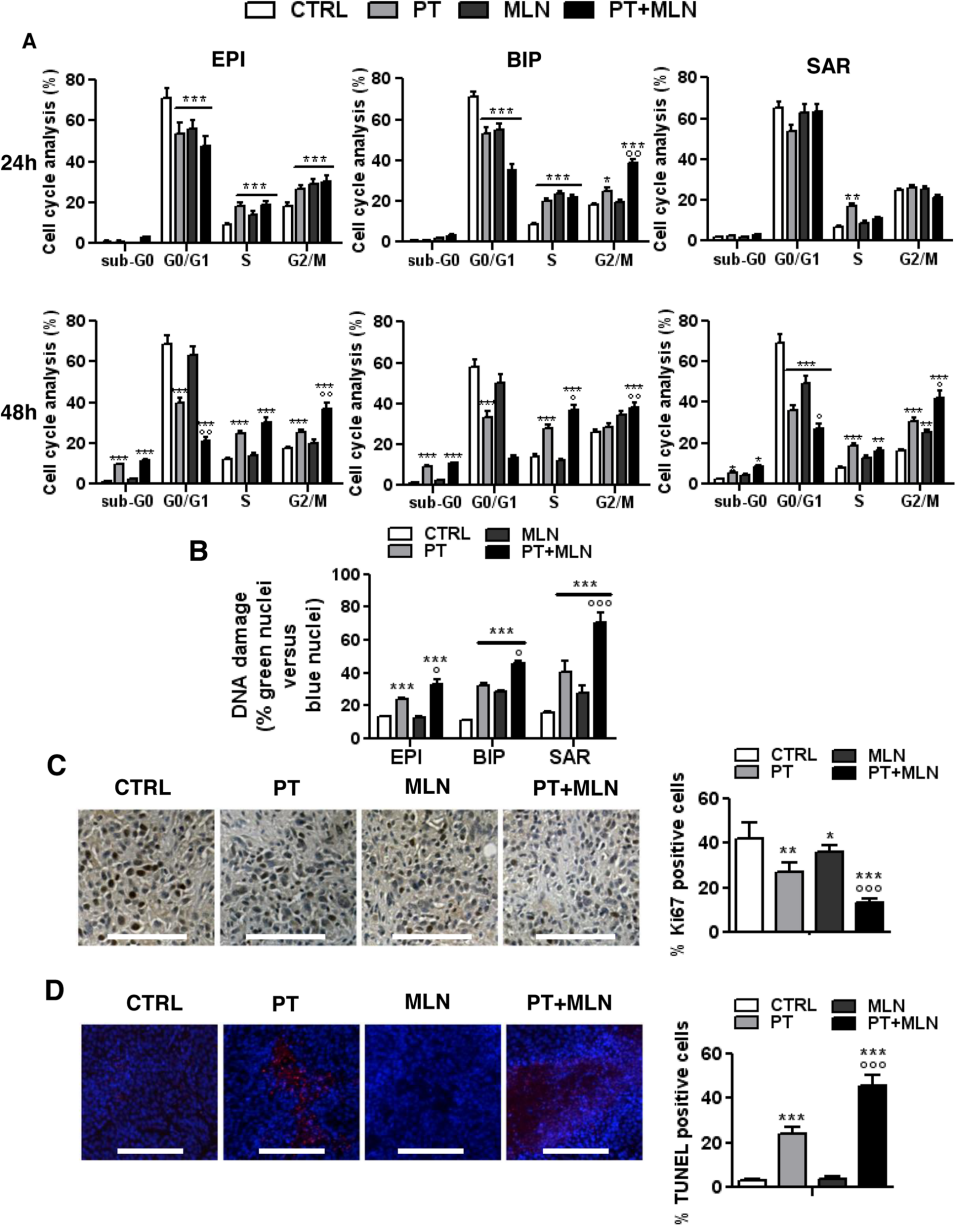
Cisplatin+MLN4924 combination induces cell cycle alteration, DNA damage and senescence. Primary MPM cells derived from 3 different histopathological subtypes, epithelioid (EPI BAP1^+^ UPN7 and EPI BAP1^-^ UPN11), biphasic (BIP BAP1^+^ UPN14 and BIP BAP1^-^ UPN16) and sarcomatous (SAR BAP1^+^ UPN22 and SAR BAP^-^ UPN23) were incubated in fresh medium (CTRL), with 50 μM cisplatin (PT), 0.2 μM MLN4924 (MLN) or their combination (PT+MLN). **A**. Cell cycle distribution was measured by flow cytometry in duplicates, after 24 h (upper panels) or 48 h (lower panels). Data are presented as means of 2 primary MPM for each histopathological subtype used + SD (*n* = 3). *p<0.05, **p<0.01, ***p < 0.001: treated cells vs CTRL cells; °*p*<0.05, °°*p* < 0.01: PT+MLN-treated cells vs PT-treated cells. **B**. DNA damage was measured fluorometrically after 24 h. Data are presented as means of 2 primary MPM for each histopathological subtype used+ SD (*n* = 3). ***p < 0.001: treated cells vs CTRL cells; °*p* < 0.05, °°°*p* < 0.001: PT+MLN-treated cells vs PT -treated cells. **C**. *Left panel*: representative sections of AB1 tumors from each group of animals, treated as indicated in Figure 1D-E, stained with the Ki67 antibody. Nuclei were counter-stained with hematoxylin. Scale bar: 100 μm (10x ocular; 40x objective). At least 10 fields were examined for each condition. *Right panel*: quantification of Ki67-positive cells versus the total number of cells, performed with the ImageJ software. Data are presented as means + SD. *p<0.05, **p<0.01, ***p < 0.001: treated cells vs CTRL cells; °°°*p* < 0.001: PT+MLN-treated cells vs PT-treated cells. **D**. *Left panel*: representative TUNEL staining on tumors sections from each group of animals, as above. Nuclei were counter-stained with DAPI. Scale bar: 200 μm. (10x ocular, 40x objective). At least 10 fields were examined for each condition. *Right panel*: quantification of TUNEL-positive cells versus the total number of cells, performed with the ImageJ software. Data are presented as means + SD. ***p < 0.001: treated cells vs CTRL cells; °°°*p* < 0.001: PT+MLN-treated cells vs PT-treated cells

Since both cisplatin [20] and MLN4924 [21] impair cell cycle progression, we firstly assessed this impairment as a possible mechanism of synergism. After 24 h, epithelioid and biphasic MPM cells were arrested in S- and G2/M-phase by cisplatin, either alone or in combination with MLN4924. MLN4924 as single agent blocked epithelioid MPM cells in S- and G2/M-phase, and biphasic MPM cells in S-phase. Sarcomatous MPM, usually associated with lower cisplatin sensitivity [1], showed only a modest increase in S-phase arrested cells in response to cisplatin (Fig. 2A, upper panels; Additional Fig. 2). After 48 h, all MPM cells treated with cisplatin plus MLN4924 showed an increase of apoptotic sub-G_0_ cells, and an arrest in S- and G2/M-phase, regardless of BAP1 status and histological subtype. Cisplatin alone had similar effects, increasing the apoptotic sub-G_0_ cells and the arrest in S- and G2/M-phase, with exception of biphasic MPM cells, where the increase in G2/M-phase was not significant. In the sarcomatous subtype MLN4924 alone increased the percentage of cells arrested in G2/M-phase, but not the percentage of cells arrested in S-phase (Fig. 2A, lower panels; Additional Fig. 3), indicating that in this subtype the kinetic of cell cycle arrest was slower. Interestingly, in sarcomatous cells (Additional Fig. 4), but not in the other subtypes (not shown), MLN4924 alone and combined with cisplatin increased the senescent phenotype. This observation may explain the different behavior of sarcomatous MPM from the other histotypes, in particular the finding that the effect of MLN4924 in sarcomatous MPM were delayed, i.e. visible at 48 h but not at 24 h. Likely, after 48 h sarcomatous cells are more senescent than epithelioid or biphasic cells, and more susceptible to cell cycle arrest.

Cisplatin also caused DNA fragmentation in all MPM histotypes, as MLN4924 did in biphasic and sarcomatous MPM. Also in this case, the drug combination was more potent than single agents in all MPM subtypes (Fig. 2B). Syngeneic MPM models recapitulated the effects observed in patient-derived cultures. Indeed, while cisplatin and MLN49 modestly decreased cells proliferation, their combination decreased more relevantly intratumor proliferation (Fig. 2C). Moreover, as indicated by the TUNEL staining, cisplatin induced intratumor apoptosis and DNA fragmentation, differently from MLN4924, but the combination treatment produced significantly stronger effects (Fig. 2D).

No changes in the autophagy flux, which we previously demonstrated as a mechanism of resistance to cisplatin in MPM [3], were induced by MLN4924 (Additional Fig. 5), leading to exclude this mechanism as potential enhancer of cisplatin toxicity.

### MLN4924 sensitizes MPM to cisplatin by inducing an ER stress-triggered immunogenic cell death

**Fig. 3:**
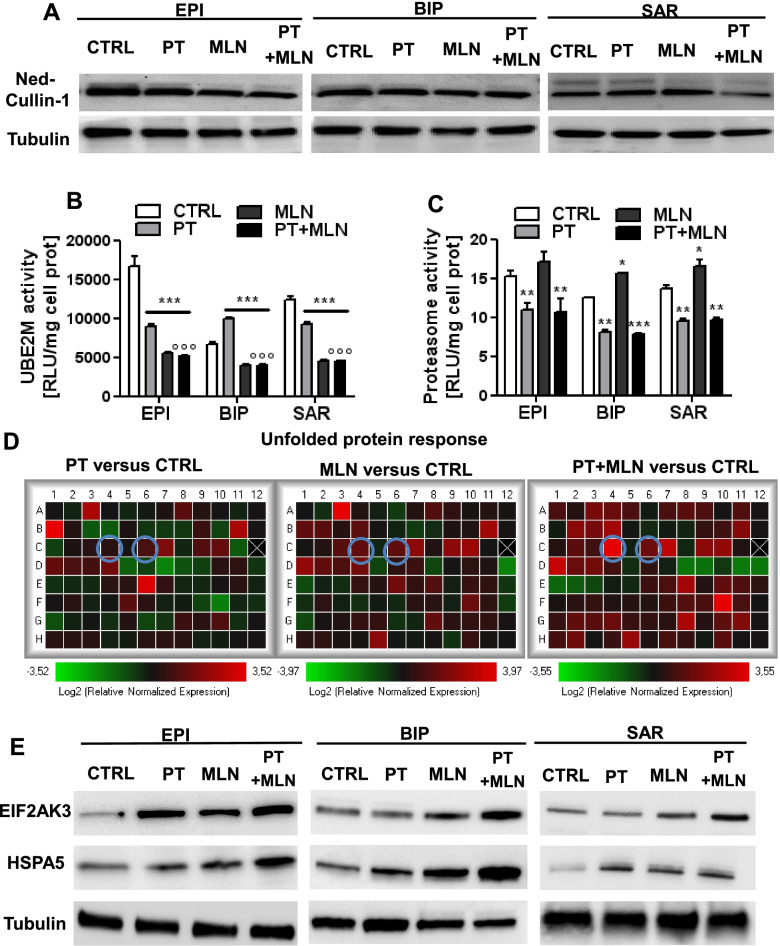
By inhibiting neddylation, MLN4924 induces a robust UPR in mesothelioma cells. Primary MPM cells derived from 3 different histopathological subtypes, epithelioid (EPI BAP1^+^ UPN7 and EPI BAP1^-^ UPN11), biphasic (BIP BAP1^+^ UPN14 and BIP BAP1^-^ UPN16) and sarcomatous (SAR BAP1^+^ UPN22 and SAR BAP^-^ UPN23) were incubated in fresh medium (CTRL), with 50 μM cisplatin (PT), 0.2 μM MLN4924 (MLN) or their combination (PT+MLN) for 24 h. In panel A and D, we showed the results of the MPM cells that resulted the top-responder cells to the cytotoxic effects of the combination of PT and MLN4924 (according to the results reported in Figure 2): epithelioid (EPI BAP1^+^ UPN7), biphasic (BIP BAP1^-^ UPN16) and sarcomatous (BAP1^+^ UPN22) MPM cells. **A**. Cullin 1 neddylation (NED, upper band) was measured by immunoblotting in. Tubulin was used as a loading control. The figure is representative of 1 out of 3 experiments with similar results on EPI BAP1^+^ UPN7, BIP BAP1^-^ UPN16 and SAR BAP1^+^ UPN22. **B**. UBE2M activity was measured spectrophotometrically in duplicates. Data are presented as means of 2 primary MPM for each histopathological subtype used + SD (*n* = 3). ***p < 0.001: treated cells vs CTRL cells; °°°*p* < 0.001: PT+MLN-treated cells vs PT-treated cells. **C**. Proteasome activity was measured fluorometrically in duplicates. Data are presented as means of 2 primary MPM for each histopathological subtype used + SD (*n* = 3). *p<0.05, **p<0.01, ***p < 0.001: treated cells vs CTRL cells. **D**. Heatmap of the Unfolded Protein Response genes relative expression, in SAR BAP1^+^ UPN 22 MPM cells. Results are expressed in a logarithmic scale. The figure is representative of 1 out of 3 experiments with similar results. Cyan circles: *EIF2AK3* and *HSPA5* genes. **E**. EIF2AK3 and HSPA5 proteins were measured by immunoblotting in epithelioid (EPI BAP1^+^ UPN7), biphasic (BIP BAP1^-^ UPN16) and sarcomatous (BAP1^+^ UPN22) MPM cells. Tubulin was used as a loading control. The figure is representative of 1 out of 3 experiments with similar results

Another well-known mechanism of cisplatin resistance in MPM is the lack of ER-triggered cell death [3, 10]. We thus analyzed if the synergistic effect of MLN4924 and cisplatin was due to the inhibition of cullin 1 neddylation and SCF complex activity: indeed, such inhibition induces the accumulation of undegraded/unfolded proteins within the ER and mounts a lethal unfolded protein response (UPR) [16]. To explore this issue, we first investigated the neddylation of cullin in the three samples that – in each MPM subtypes – resulted the top-responder to the cytotoxic effects of the MLN4924 and cisplatin combination (Fig. 3A). In all the MPM subtypes, MLN4924 decreased the amount of neddylated cullin 1, while cisplatin had no effect (Fig. 3A). Expanding the analysis to additional MPM cells, including at least two MPM for each histotype, one BAP1^+^ and one BAP1^-^ MPM, we found that MLN4924 decreased activity of UBE2M (Fig. 3B), a component of the enzymatic system required for cullin 1 neddylation [22]. While MLN4924 impaired the neddylation/UBE2M system, it only slightly increased proteasome activity (Fig. 3C). Conversely, cisplatin had mixed effects on UBE2M activity among the different MPM subtypes (i.e. increase of UBE2M activity in epithelioid and sarcomatous MPM, decrease in biphasic MPM; Fig. 3B), but it always decreased proteasome activity (Fig. 3C). All the effects on cullin 1 neddylation, UBE2M activity and proteasome activity, exerted by the single agents, were maintained by their combination (Fig. 3A-C).

To prove that this combination increased the burden of unfolded proteins to the threshold sufficient to elicit an ICD-mediated cell death, we analyzed the transcriptomic profile of 84 genes involved in UPR (Fig. 3D; Additional Table 4), in a sarcomatous MPM that was one of the top responders to the MLN4924 and cisplatin combination, but showed high resistance to cisplatin (Fig 1A-C; Additional file 1). UPR sensors and executers were progressively up-regulated in MPM cells treated with cisplatin alone, MLN4924 alone or their combination (Fig 3D). Two of the first executers of UPR-dependent cell death [23] - HSPA5/BiP/GRP78 and EIF2AK3/PERK - were among the top-up-regulated genes (Additional Table 4) as mRNA (Fig. 3D) and protein (Fig. 3E) in the top-responder cells to the cytotoxic effects of the combination of MLN4924+cisplatin (Fig. 2), in each MPM subtype (Fig. 3E). Once again, the increase in EIF2AK3 and HSPA5 protein elicited by the cisplatin or MLN4924 alone, was enhanced by their combination (Fig. 3E).

**Fig. 4:**
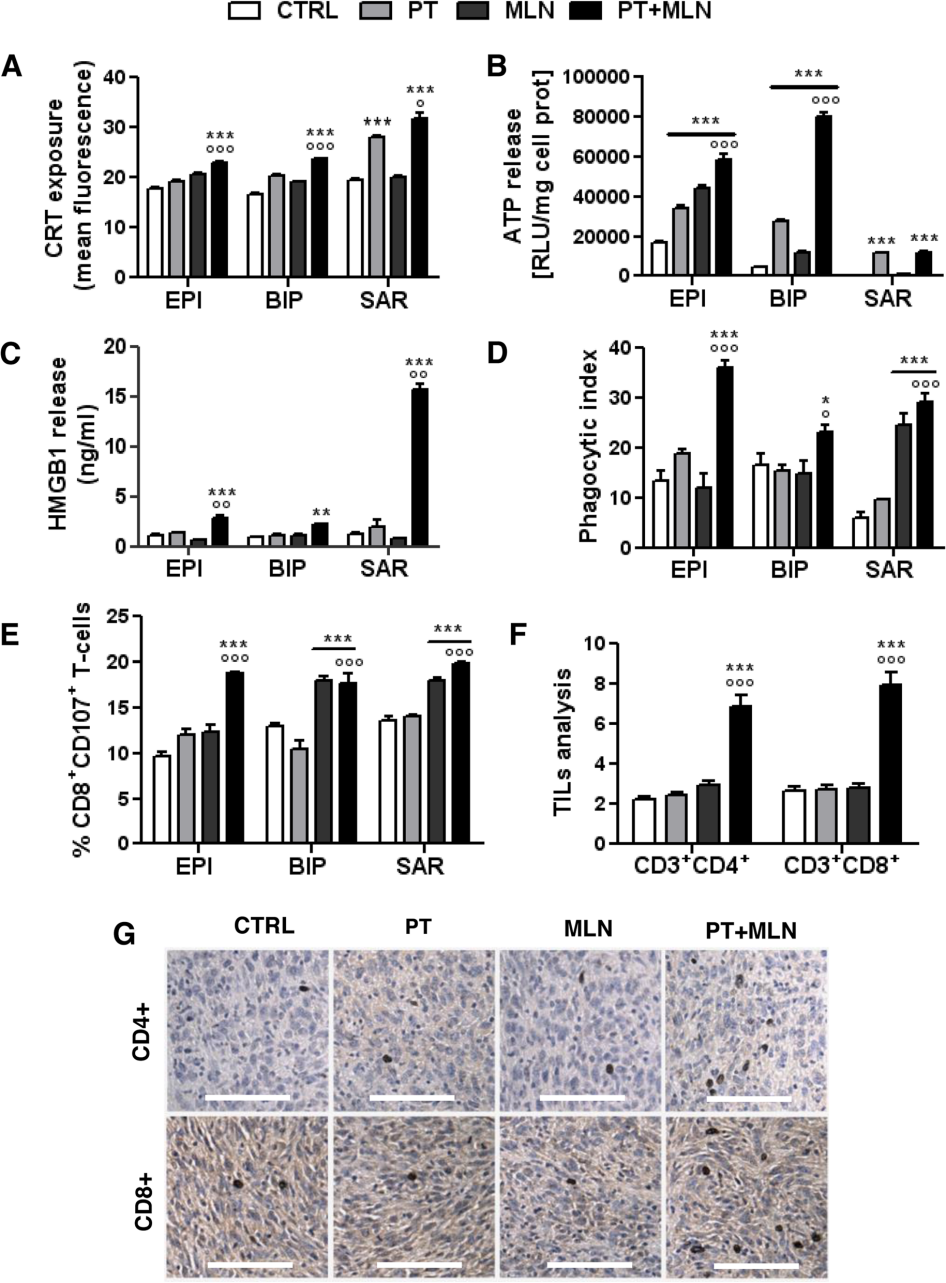
MLN4924 in combination with cisplatin effectively restored immunogenic cell death in resistant mesothelioma cells. Primary MPM cells derived from 3 different histopathological subtypes, epithelioid (EPI BAP1^+^ UPN7 and EPI BAP1^-^ UPN11), biphasic (BIP BAP1^+^ UPN14 and BIP BAP1^-^ UPN16) and sarcomatous (SAR BAP1^+^ UPN22 and SAR BAP^-^ UPN23) were incubated in fresh medium (CTRL), with 50 μM cisplatin (PT), 0.2 μM MLN4924 (MLN) or their combination (PT+MLN) for 24 h Panels A-B) or 48 h (panel **C**). **A**. Calreticulin (CRT) exposure on the plasma membrane was measured by flow cytometry in duplicates. Data are expressed as means of 2 primary MPM for each histopathological subtype used + SD (*n* = 3). ***p < 0.001: treated cells vs CTRL cells; °*p* < 0.05, °°°*p* < 0.001: PT+MLN-treated cells vs PT-treated cells. **B**. ATP release was measured by a chemiluminescence-based assay in duplicates. Data are expressed as means of 2 primary MPM for each histopathological subtype used + SD (*n* = 3). ***p < 0.001: treated cells vs CTRL cells; °°°*p* < 0.001: PT+MLN-treated cells vs PT-treated cells. **C**. HMGB1 release was measured by ELISA in duplicates. Data are expressed as means of 2 primary MPM for each histopathological subtype used + SD (*n* = 3). **p < 0.01, ***p < 0.001: treated cells vs CTRL cells; °°*p* < 0.01: PT+MLN-treated cells vs PT-treated cells. **D**. Phagocytosis by dendritic cells was measured by flow cytometry in duplicates. Data are expressed as means of 2 primary MPM for each histopathological subtype used + SD (*n* = 3). *p < 0.05, ***p < 0.001: treated cells vs CTRL cells; °*p* < 0.05, °°°*p* < 0.001: PT+MLN-treated cells vs PT-treated cells. **E**. CD8^+^CD107a^+^ lymphocytes, collected after co-culture with dendritic cells that have phagocytized MPM cells, were quantified by flow cytometry, in duplicates. Data are expressed as means of 2 primary MPM for each histopathological subtype used+ SD (*n* = 3). ***p < 0.001: treated cells vs CTRL cells; °°°*p* < 0.01: PT+MLN-treated cells vs PT-treated cells. **F-G**. AB1 tumors were subcutaneously implanted into 6-week-old female BALB/c mice when the tumor size reached 50 mm^3^ mice (*n* = 10 animals/group) were treated for 3 consecutive as indicated in the Methods section. **F**. Percentage of CD4^+^/CD8^+^ intratumor T-lymphocytes (TILs) collected after tumor digestion and analyzed by flow cytometry, in duplicates. Data are means + SD. ***p < 0.001: treated group vs CTRL group; °°°*p* < 0.001: PT+MLN-treated cells vs PT-treated cells. **G**. Representative immunohistochemical imaging of CD4^+^ and CD8^+^ TILs. Scale bar: 100 μm. (10x ocular, 40x objective). At least 10 fields were examined for each condition

Being a DNA damaging agent (Fig. 2B-D) but a poor inducer of ER stress (Fig. 3D; Additional Table 4), cisplatin produced only modest effects or no effects on CRT exposure and HMGB1 release. The only ICD parameters significantly increased were the surface levels of CRT in sarcomatous MPM cells and the release of ATP in all histotypes (Fig. 4A-C). These changes, however, were not sufficient to induce a complete ICD, because cisplatin did not increase the MPM cells phagocytosis by DCs (Fig. 4D) and the subsequent expansion of activated CD8^+^CD107a^+^T-cells, endorsed with anti-tumor activity (Fig. 4E). MLN4924, a lower inducer of DNA damage (Fig. 2B-D) but a stronger inducer of ER stress (Fig. 3D; Additional Table 4), also produced mild effects on ICD parameters: it did not significantly increase CRT exposure and HMGB1 release, but it increased the ATP leakage, except in the sarcomatous MPM cells (Fig. 4A-C). Despite these poor and highly variable results obtained by the single agents, their combination increased the ICD followed by MPM cells phagocytosis and expansion of CD8^+^CD107a^+^T-cells in all the MPM subtypes (Fig. 4A-E, Additional Fig. 6). Interestingly, the percentage of CD8^+^CD107a^+^T-cells doubled from 10% in the basal condition (untreated MPM cells phagocytized by DCs) to 20% in the condition of MPM cells treated with the combination of cisplatin and MLN4924, indicating that a larger proportion of CD8^+^T-cells acquired cytolytic properties after this treatment. The remaining T-cells were likely no-cytolytically activated CD8^+^T-cells, or CD4^+^T-cells, natural killer cells, T-regulatory cells, as observed in previous studies where immune-phenotyping of the T-cell population showed immune cells which were in expansion in the presence of MPM cells [5].

Relevantly, the combination of cisplatin and MLN4924 achieved a good activation of the host immune system in syngeneic MPM models, as it was the only treatment that significantly increased the percentage of CD4^+^ and CD8^+^ TILs (Fig. 4F-G).

Interestingly, sarcomatous MPMs, which were usually poorly responsive to cisplatin [1], were the more vulnerable to the ICD elicited by the combination therapy. To explain the different response among subtypes, we measured the TMB that may generate neoantigens derived from DNA damage, able to activate T-lymphocytes. However, we did not detect significant differences in TMB between histotypes and treatments (Additional Table 5), suggesting that the differential ICD elicited by cisplatin plus MLN4924 likely relies on a differential UPR-related activity more than on differential DNA damage and TMB.

### Mesothelioma cells highly expressing SKP2 are more sensitive to MLN4924

**Fig. 5:**
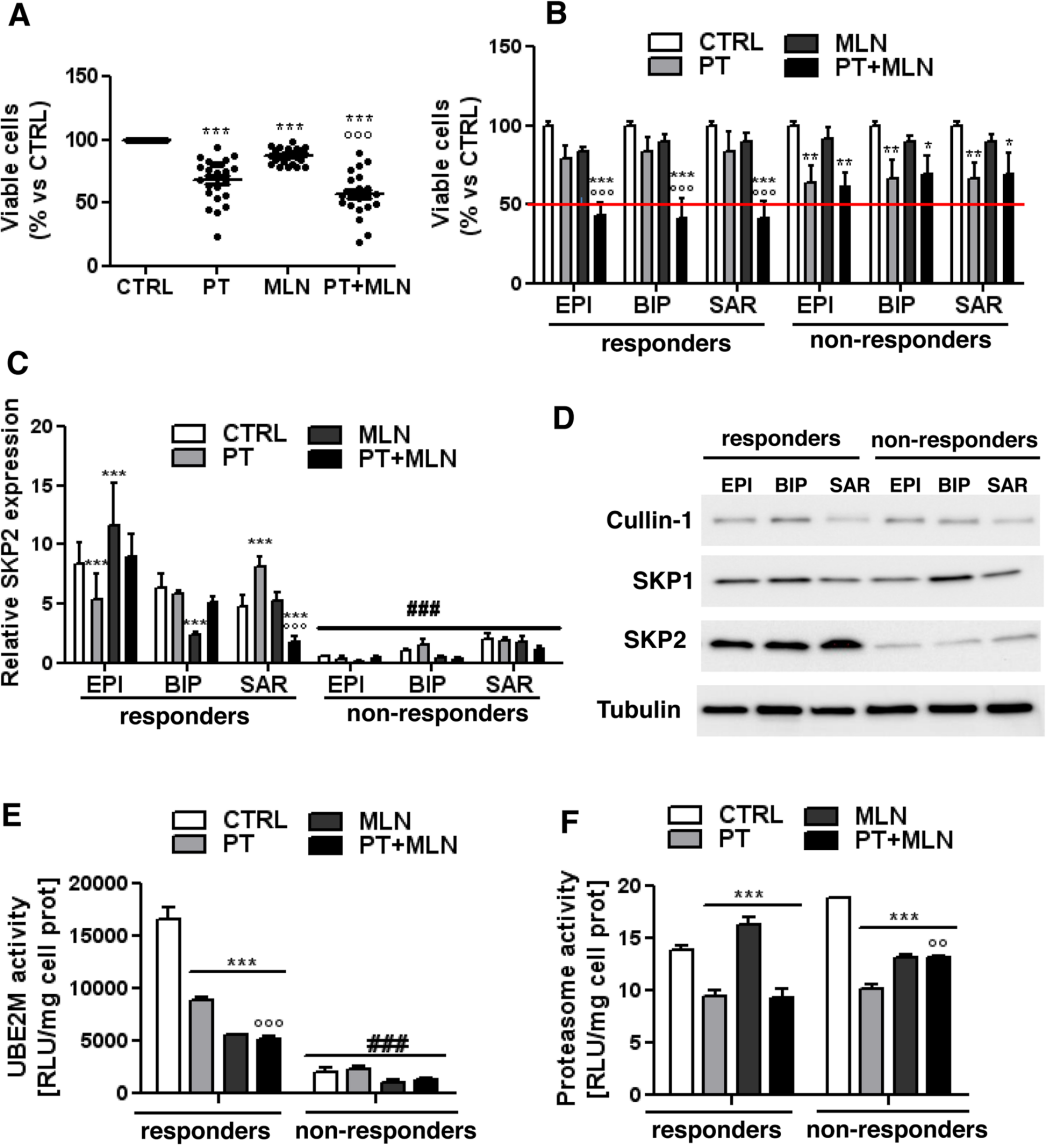
MLN4924-responsive cells have higher levels of SKP2. **A-B**. Primary mesothelioma cells of epithelioid (EPI; *n*= 10), biphasic (BIP; *n* = 5), sarcomatous (SAR; *n* =4) origin were incubated 72 h in fresh medium (CTRL), with 50 μM cisplatin (PT), 0.2 μM MLN4924 (MLN) or their combination (PT+MLN). Crystal violet staining was quantified spectrophotometrically. **A**: disaggregated data of cell viability. **B**: MPM cells divided in responders (samples where the combination of cisplatin+MLN4924 decreased the number of viable cells below 50%) and non-responders (samples where the combination of cisplatin+MLN4924 maintained >50% viable cell, red line). Data are presented as means + SD; each sample was analyzed in quadruplicates. * *p* < 0.05, **p < 0.01, ***p < 0.001: treated cells vs CTRL cells; °°°*p* < 0.001: PT+MLN-treated cells vs PT -treated cells. **C-F**. Top responder (the epithelioid EPI BAP1^+^ MPM UPN7, the biphasic BIP BAP1^-^ MPM UPN16, the sarcomatous SAR BAP1^+^ MPM UPN22) and top non-responder (the epithelioid EPI BAP1^+^ MPM UPN6, the biphasic BIP BAP1^+^ MPM UPN18, the sarcomatous SAR BAP1^-^MPM UPN21) cells were incubated as in **A-B** for 24 h **C.** SKP2 mRNA levels were measured by RT-PCR, in triplicates. Data are presented as means + SD (*n* = 3). ***p < 0.001: treated cells vs CTRL cells, °°°*p* < 0.001: treated cells vs PT-treated cells; ^###^*p* < 0.001: non-responder cells vs respective responder cells. **D**. The expression of SCF complex proteins – cullin 1, SKP1 and SKP2 - was measured by immunoblotting. Tubulin was used as a loading control. The figure is representative of 1 out 3 experiments with similar results. **E**. UBE2M activity was measured spectrophotometrically, in duplicates. Data are presented as means + SD (*n* = 3). ***p < 0.001: treated cells vs CTRL cells, °°°*p* < 0.001: treated cells vs PT-treated cells; ^###^*p* < 0.001: non-responder cells vs respective responder cells. **F**. Proteasome activity was measured fluorometrically, in duplicates. Data are presented as means + SD (*n* = 3). ***p < 0.001: treated cells vs CTRL cells; °°*p* < 0.01: treated cells vs PT-treated cells

To correlate the sensitivity of cisplatin plus MLN4924 with specific molecular features of MPM patients related to the SCF complex, from our cohort of 23 MPM cultures derived from patients, we selected the samples from 19 patients receiving carboplatin or cisplatin as first-line treatment (Additional Table 1), and we first tested the efficacy of the combination of cisplatin and MLN4924 in this larger cohort of patients (Fig. 5A). Although the mean efficacy of cisplatin plus MLN4924 combination was significantly higher than the efficacy of the single agents, we noticed that in 6 MPM patient-derived cells the addition of MLN4924 did not enhance cisplatin cytotoxicity (Fig. 5A). These samples were defined as non-responder MPM cells, defined as samples where the combination of cisplatin+MLN4924 maintained >50% viable cells. The responder MPMs were defined as samples where the combination of cisplatin plus MLN4924 decreased the number of viable cells below 50%. Responders and non-responder MPMs were equally distributed among the three subtypes (Fig. 5B) and BAP1 mutational status (Additional Table 2). We chose the 3 top responder patients (epithelioid BAP1^+^ MPM UPN7, biphasic BAP1^-^ MPM UPN16, sarcomatous BAP1^+^ MPM UPN22) and the 3 top non-responders (epithelioid BAP1^+^ MPM UPN6, biphasic BAP1^+^ MPM UPN18 and sarcomatous BAP1^-^MPM UPN21), to further clarify the key molecular determinants of this differential sensitivity. To exclude inter-histotype differences, we took care to select one MPM per each histotype.

The sensitivity was independent from the *Cul1* mutational status: indeed, both responders and non-responders harbored the wild-type *Cul1* alleles (Additional Fig. 7A). About the SCF complex, neither cullin-1 and SKP1 mRNA (Additional Fig. 7A-B) nor protein levels (Fig. 5D) distinguished responder from non-responder patients. By contrast, responder MPM had higher SKP2 mRNA (Fig. 5C) and protein (Fig. 5D) than non-responder MPMs. Consistently with the higher levels of SKP2, responder MPM cells had higher UBE2M activity, which was down-regulated by MLN4924, either alone or in combination with cisplatin (Fig. 5E). Conversely, non-responder cells had low UBE2M activity that was unchanged with the treatments. Proteasome activity was comparable between responders and non-responders, and it was similarly decreased by cisplatin in both groups (Fig. 5F). The addition of MLN4924 did not further change proteasome activity in responder MPM cells, but interestingly cisplatin increased proteasome activity in non-responder cells (Fig. 5F).

### SKP2 level dictates sensitivity to MLN4924 and resistance to cisplatin, and is a prognostic factor in mesothelioma patients

**Fig. 6:**
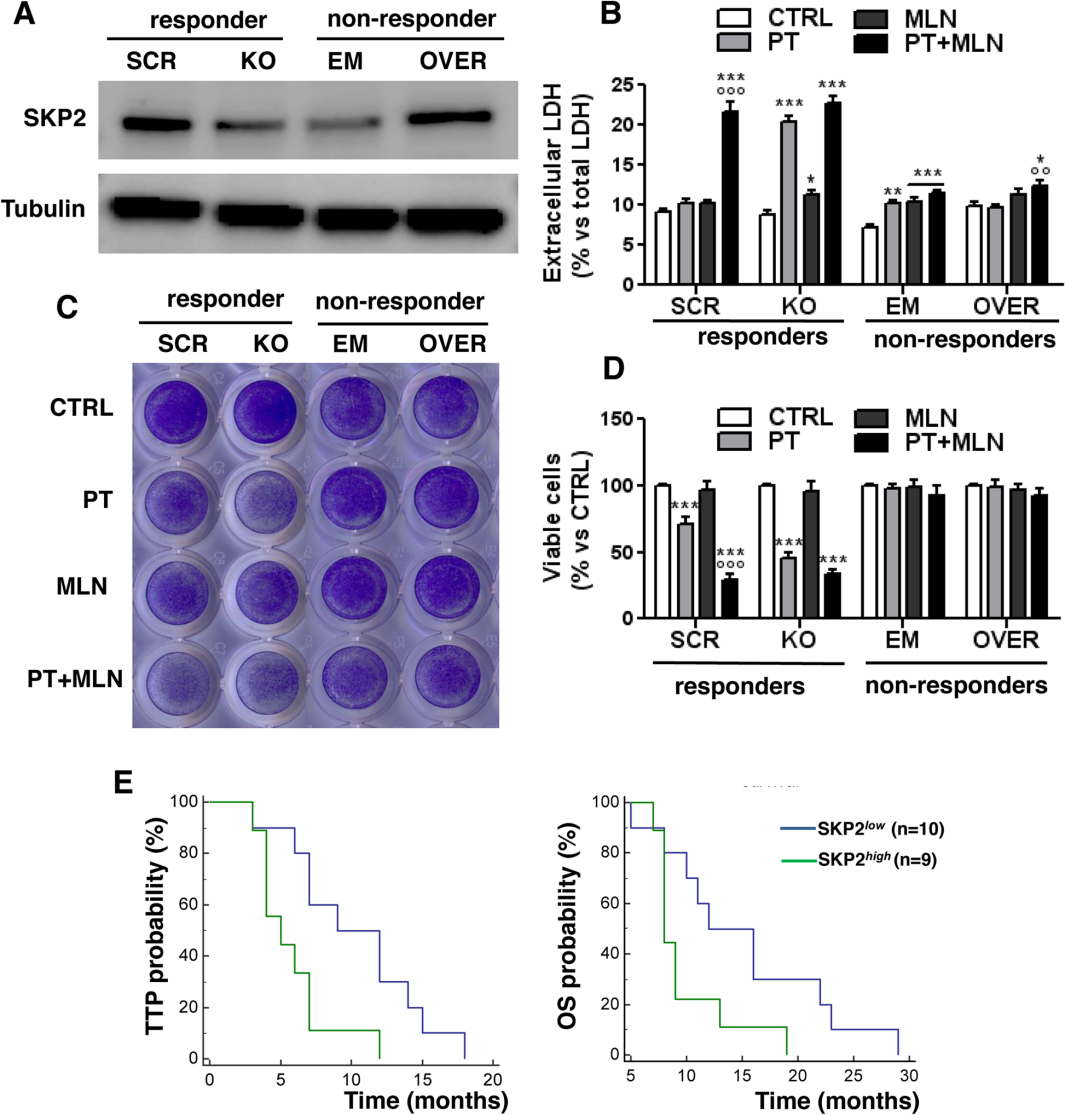
SKP2 levels controls the sensitivity to cisplatin and MLN4924 in malignant pleural mesothelioma cells. Top responder cells (EPI BAP1^+^ MPM UPN7) to the combination cisplatin+MLN4924 were transduced with a non-targeting scrambled vector (SCR) or with a CRISPR/Cas9 SKP2-knocking out vector (KO). Top non-responder cells (EPI BAP1^+^ MPM UPN6) were transduced with an empty vector (EM) or with an expression vector for SKP2 (OVER). **A**. SKP2 expression was measured by immunoblotting. Tubulin was used as a loading control. The figure is representative of 1 out of 3 experiments with similar results. **B-D**. Cells were incubated for 48 h (panel **B**) or 72 h (panel **C-D**) in fresh medium (CTRL) with 50 μM cisplatin (PT), 0.2 μM MLN4924 (MLN) or their combination (PT+MLN). **B**. LDH release, taken as cytotoxicity index, was measured spectrophotometrically in triplicates. Data are presented as means + SD (*n* = 3). *p < 0.05, **p < 0.01, ***p < 0.001: treated cells vs CTRL cells; °°*p* < 0.01, °°°*p* < 0.001: PT+MLN-treated cells vs PT -treated cells. **C**. Representative photographs of crystal violet staining, from 1 of 3 experiments. **D**. Crystal violet staining was quantified spectrophotometrically. Data are presented as means + SD; each sample was analyzed with technical quadruplicates. ***p < 0.001: treated cells vs CTRL cells; °°°*p* < 0.001: PT+MLN-treated cells vs PT -treated cells. **E**. SKP2 expression was measured by RT-PCR in samples of each patient (n=19) receiving platinum-derivatives as first-line treatment, and median value was calculated. Patients were classified as SKP2^low^ (blue line, including 10 patients) and SKP2^high^ (green line, including 9 patients) if the mRNA levels were low or equal/higher than the median value. Time to progression (TTP; *left panel*) and overall survival (OS; *right panel*) probability was calculated using the Kaplan-Meier method. *p<0.016 (TTP); *p<0.044 (OS): SKP2^high^ vs SKP2^low^ group

To prove the role of SKP2 as key determinant of sensitivity to SCF complex inhibitors, we knocked out SKP2 in the three top responder MPMs (UPN7, UPN16, UPN22) and we overexpressed it in the three non-responder MPMs (UPN6, UPN18, UPN21). UPN7, i.e. one top responder MPM with high levels of endogenous SKP2, and UPN6, i.e. one top non-responder MPM with the lowest level of SKP2, showed the highest efficacy of SKP2 knocked-out and overexpression (Fig. 6A), and were chosen for the following proof of concept assays to demonstrate the role of SKP2 as determinants of sensitivity or resistance to the MLN4924 and cisplatin combination. SKP2-knocked-out MPM were more sensitive to cisplatin compared to untreated cells, as demonstrated by the increased necrosis indicated by the LDH release (Fig. 6B) and the decreased viability (Fig. 6C-D). SKP2 knocked-out cells did not have different sensitivity to MLN4924 compared to control cells, and did not show further benefits from the combination of MLN4924 and cisplatin comparing to cisplatin alone (Fig. 6B-D). By contrast, SKP2-overexpressing MPM cells displayed higher resistance to cisplatin compared to their control cells. MLN4924 alone had no effect on non-responder cell overexpressing SKP2. Only the combination of MLN4924 and cisplatin increased the LDH release, resulting more effective than the single agents (Fig. 6B), but it did not decrease the cell viability compared to untreated cells or to cells treated with the single agents (Fig. 6C-D). This result suggested that the overexpression of SKP2 in MPM cells non-responding to the MLN4924 + cisplatin combination was only partially benefited by the combinatorial treatment. On the other hand, we speculated that high levels of SKP2 could be indicative of resistance to cisplatin in clinical settings. To prove this hypothesis, we stratified the patients of our cohort treated with cisplatin or carboplatin as first-line treatment according to the median level of SKP2 in the tumours. Indeed, SKP2^*low*^ group showed a significantly higher TTP, suggesting a better response to platinum therapy, and higher overall survival (OS) than the SKP2^*high*^ group (Fig. 6E).

## Discussion

Previous experimental evidence suggested that targeting the ubiquitination system may be a promising strategy to restore cisplatin efficacy in MPM at preclinical levels [3, 24], but proteasome inhibitors had disappointing results in clinical trials [11]. To make a step forward and look for a more specific agent than proteasome inhibitor, we investigated the first-in-class neddylation inhibitor MLN4924, which showed a strong synergistic profile with cisplatin, associated with good safety, in our patient-derived MPM cultures and animal models.

Differently from proteasome inhibitors that showed cytotoxicity as single agents [3, 24], MLN4924 did not. This difference could be due to the narrower number of proteins target of MLN4924 compared to proteasome inhibitors. Indeed, MLN4924 only inhibits the E3-ubiquitin ligase SCF complex, that ubiquitinates a limited number of specific proteins [16], including many onco-suppressive proteins inhibiting the progression in cell cycle [21]. The rationale of using MLN4924 as anti-tumor agent is explained by the inhibition of the SCF complex and by the consequent accumulation of oncosuppressive proteins that decrease the cell proliferation rate.

MLN4924, currently under investigation in several clinical trials, alone or associated with other cytotoxic drugs (https://clinicaltrials.gov), has increased cisplatin efficacy against pancreatic [21], ovarian [25], bladder [26] and esophageal [27] cancer. Our study supports the potential clinical investigation of MLN4924 in MPM, where an effective treatment alternative to the first-line treatment based on cisplatin-pemetrexed is lacking.

The synergism with cisplatin, confirmed by the very low combination index, suggests that both drugs target common pathways. Indeed, cisplatin and MLN4924 are known hampering the cell cycle progression with different mechanisms: cisplatin induces DNA damage [20], MLN4924 prevents the degradation of cyclin-dependent kinase inhibitor p21^WAF1^, cyclin-dependent kinase inhibitor 1B (CDKNB1/p27), *WEE1* G2 checkpoint kinase, cyclin B, chromatin licensing and DNA replication factor 1 (CDT1) [21]. MLN4924-treated esophageal cancer cells showed a block in the S-phase, leading to apoptosis and/or senescence [27]. The same events are induced by cisplatin [20]. The inhibitory effects of both MLN4924 and cisplatin on cell cycle progression explain one possible mechanisms of synergism between the drugs. In MPM, the cell cycle arrest elicited by the combination was delayed also in the sarcomatous subtype after 48 h. Moreover, no significant differences were observed among MPMs with different BAP1 mutational status. This result is relevant since sarcomatous MPM with mutated/deleted BAP1 has usually the worst response to cisplatin alone [1].

Analyzing cell cycle-independent effects, we found that MLN4924 prevented the UBE2M-dependent neddylation of cullin 1, as recently observed in renal cell carcinoma [28], while cisplatin induced a strong decrease in proteasome activity, already reported in non-small cell lung cancer cells [29]. Both these mechanisms increase the number of unfolded proteins that cannot be eliminated [23]. While the proteostatic stress elicited by cisplatin or MLN4924 as single agent was mild, their combination boosted the UPR-triggered cell death because such combination blocks two sequential steps in the protein degradation pathways, i.e. ubiquitination (with MLN4924) and proteasomal activity (with cisplatin). This sequential block provides a second explanation for the synergistic effect between MLN4924 and cisplatin.

The accumulation of unfolded proteins is a classical trigger of ER stress and ICD [7]. The ability of cisplatin to induce ER stress by inhibiting the proteasomal activity is low and the ability to trigger ICD is controversial and tumor dependent [30, 31]. In our experimental conditions, the ER stress elicited by cisplatin induces some ICD-related parameters as the release of ATP, not followed by the MPM cells phagocytosis and the expansion of anti-tumor CD8^+^T-lymphocytes.

MLN4924 has been reported to cause ICD in colorectal cancers with deficient DNA mismatch repair, characterized by hyper-mutations and proteome instability, compensated by the increased activation of neddylation pathway. In this setting, the inhibition of the SCF complex promotes the induction of ICD, particularly in combination with immune-checkpoint inhibitors [32]. This mechanism does not seem the case of MPM that, differently from colorectal cancer, is characterized by a low genomic instability [33]. Indeed, neither cisplatin nor MLN4924, alone or combined, increased the TMB, notwithstanding they both induce DNA damage. The significant ICD observed *ex vivo* in patient-derived MPM cells and in mice treated with the combination of MLN4924 and cisplatin was not likely due to the accumulation of genomic lesions that produce the appearance of neoantigens. Most likely, ICD was induced by the accumulation of unfolded and undegraded proteins that induce a lethal ER stress, promoted by the inhibition of both SCF complex and proteasome inhibition by each agent.

With the aim of identifying patients who maximally benefit from the combination, we investigated 19 primary MPM cultures derived from patients treated with platinum-derivatives as first-line treatment. About 75% of the cultures displayed a reduction of cell viability >50% when treated with the combination of cisplatin and MLN4924 and were defined as responder MPMs, while for the other 25% of cases MLN4924 did not add any benefit compared to cisplatin alone. This subset was termed non-responder to the combination of MLN4924 and cisplatin. The sensitivity to the combinatorial treatment was dictated by the levels of SKP2, a F-box protein that is an integral part of SCF complex [16] with a well-defined role in tumorigenesis, invasion, metastasis and angiogenesis [34, 35]. Recently, SKP2 has been demonstrated to confer resistance to multiple chemotherapeutic drugs, making it an intriguing pharmacological target in chemo-resistant tumors [36].

In melanoma cells, the efficacy of MLN4924 has been correlated with the ability of preventing the degradation of p21^WAF1^, a known substrate of SKP2 [37], providing an indirect demonstration of a link between the levels of SKP2 and the efficacy of MLN4924. In MPM the landscape is more complex, because we discovered that SKP2 regulates the sensitivity to both cisplatin alone and cisplatin+MLN4924 combination. SKP2 knockout and overexpression demonstrated that low levels of SKP2 were associated with higher sensitivity to cisplatin compared to wild-type cells, but in these cells the MLN4924+cisplatin combination was not superior to the cisplatin alone. We hypothesize that the low levels of SKP2, one of the main target of MLN4924, reduces the benefits derived from adding the latter to cisplatin.

Accordingly, samples with high levels of SKP2 were the most responsive to the combination of MLN4924 and cisplatin. The exogenous overexpression of SKP2 in non-responder patients, however, produced only modest effects in terms of increased necrosis after the combination treatment, not followed by a decreased viability. Since SKP2 works in cooperation with the other component of the SCF complex, we may speculate that the endogenous levels of SKP2, which influences the activity of SCF complex, are the true determinant of the sensitivity toward the MLN4924 and cisplatin that disrupts both SCF complex and proteome activity. The introduction of supra-physiological levels of SKP2, not accompanied by a parallel increase of the other components of the SCF complex, does not guarantee and increase sensitivity to MLN4924. Notably, however, SKP2 overexpressing cells are completely resistant to cisplatin. The clinical implication of this observation was verified in the cohort of MPM patients treated with platinum derivatives as first line treatment. This retrospective analysis indicated for the first time that SKP2 determines poor response to cisplatin in MPM patients.

**Fig. 7:**
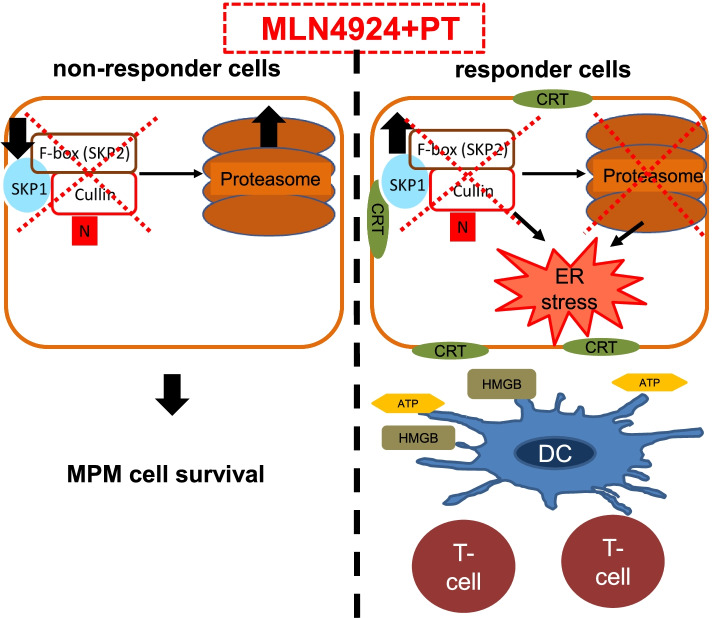
Mechanism of cisplatin MLN4924 combination action in MPM cells. MLN4924-non-responsive cells escape the lethal UPR because the low levels of UBE2M and SKP2, and the high activity of proteasome even in the presence of cisplatin prevents the accumulation of unfolded proteins and the consequent immunogenic cell death (ICD). By contrast, MLN4924-responsive cells had higher SKP2 levels and UBE2M activity, coupled with low proteasome activity when treated with cisplatin. The simultaneous block of SCF complex and proteasome enormously increases the accumulation of unfolded/ubiquitinated protein, triggering ER stress, exposure of calreticulin (CRT) on the cell surface, release of ATP and high mobile group B1 (HMGB1), activation of dendritic cells (DC) and CD8^+^T-cell against tumor, therefore inducing a canonical ICD. N: NEDD8.

Overall, we propose that MPM cells responsive to the MLN4924+cisplatin combination have physiologically higher SKP2 levels and UBE2M activity, coupled with low proteasome activity in response to cisplatin. This phenotype favors the sensitization to MLN4924+cisplatin combinations, which sequentially impairs the ubiquitination and the proteasome degradation of proteins. This “two-hits” mechanism causes a massive accumulation of unfolded proteins and triggers the consequent ER stress-triggered ICD (Fig. 7). On the other hand, low levels of the physiological targets of MLN4924 – SKP2 and UBE2M – limit the efficacy of SCF complex inhibitors, preventing the accumulation of unfolded proteins and the UPR-dependent ICD.

## Conclusions

In conclusion, we have demonstrated that neddylation inhibitor MLN4924 may improve cisplatin efficacy in MPM. The two drugs exert strongly synergistic effects inducing decrease in tumor growth, proliferation and viability caused by DNA damage and induction of a proteostatic stress, followed by UPR-mediated apoptosis and ICD. Based on the mechanism of synergism identified, we propose that the association of neddylation inhibitors and cisplatin should be clinically investigated in patients non-responsive to platinum-based chemotherapy.

Moreover, we identified SKP2 as a marker with a double predictive potential: high levels of SKP2 predict a worse response to the first-line cisplatin-based chemotherapy, but they suggest at the same time the maximal potential benefit achieved by the combination of cisplatin and MLN4924. We suggest considering SKP2 as a potential stratification marker that provides useful information on the efficacy of platinum-derivatives treatments and on the benefits derived from the inclusion of ubiquitination inhibitors in the platinum-based treatment protocols for MPM patients.

## Supplementary Information

**Additional file 1.**

## Data Availability

All data generated or analysed during this study are included in this published article [and its supplementary information files].

## Abbreviations

BiP: Binding Immunoglobulin Protein
CDKNB1 or p27: Cyclin-dependent kinase inhibitor 1B
CDT1: Chromatin licensing and DNA replication factor 1
CI: The Combination Index
CRT: Calreticulin
DCs: Dendritic cells
EIF2AK3: Eukaryotic Translation Initiation Factor 2 α Kinase 3
ER: Endoplasmic reticulum
GRP78: Glucose-Regulated Protein 78kDa
HMGB1: high mobility group protein
HSP5A: Heat Shock 70kDa Protein 5
ICD: Immunogenic cell death
LDH: Lactate dehydrogenase
MPM: Malignant pleural mesothelioma
NEDD8: Neural precursor cell expressed developmentally down-regulated 8
OS: Overall survival
p21: Cyclin-dependent kinase inhibitor 1
PERK: Protein kinase R-like endoplasmic reticulum kinase
PFS: Progression free survival
SCF: SKP/Cullin/F-box
SKP: S-Phase Kinase Associated Protein
TILs: Tumor infiltrating lymphocytes
TMB: Tumor Mutational Burden
TTP: Time to progression
UBE2M: Ubiquitin-conjugating enzyme E2M
