# Supplementary material for: SKP2 drives the sensitivity to neddylation inhibitors and cisplatin in malignant pleural mesothelioma
Source: J Exp Clin Cancer Res. 2022 Feb 23;41:75. doi: 10.1186/s13046-022-02284-7 (PMC8864928; doi:10.1186/s13046-022-02284-7)
Supplement: Supplementary file 1 — Additional file 1. [file 13046_2022_2284_MOESM1_ESM.docx]

**Supplementary materials**

**Additional Table 1. Anamnestic and clinical features of the patients**

| **UPN** | **Histotype** | **Gender** | **Age** | **Asbestos exposure** | **First line**  **treatment** | **Second line treatment** | **TTP**  **(months)** | **OS**  **(months)** |
| --- | --- | --- | --- | --- | --- | --- | --- | --- |
| 1 | Epithelioid | M | 78 | Possible | No | No | 3 | 6 |
| 2- | Epithelioid | M | 69 | Yes | No | ­No | 2 | 12 |
| 3 | Epithelioid | M | 53 | Yes | No | ­No | 2 | 13 |
| 4 | Epithelioid | M | 44 | Yes | Carbo+Pem | Gem+ Vin | 14 | 29 |
| 5 | Epithelioid | M | 79 | Possible | Carbo+Pem | Gem | 12 | 16 |
| 6 | Epithelioid | M | 68 | Yes | Carbo+Pem | Pem | 4 | 9 |
| 7 | Epithelioid | M | 76 | Unknown | CisPt+Pem | No | 3 | 8 |
| 8 | Epithelioid | M | 74 | Unknown | Carbo+Pem | No | 7 | 11 |
| 9 | Epithelioid | M | 58 | Yes | Carbo+Pem | Pem | 6 | 13 |
| 10 | Epithelioid | F | 84 | Yes | CisPt+Pem | No | 7 | 8 |
| 11 | Epithelioid | M | 62 | Yes | Carbo+Pem | No | 12 | 16 |
| 12 | Epithelioid | F | 46 | Yes | Carbo+Pem | Nintedanib | 9 | 12 |
| 13 | Epithelioid | M | 54 | Unknown | CisPt+Pem | No | 4 | 8 |
| 14 | Biphasic | M | 53 | Yes | Carbo+Pem | Carbo+Pem | 12 | 19 |
| 15 | Biphasic | M | 60 | Yes | Carbo+Pem | Gem+Vin | 18 | 23 |
| 16 | Biphasic | M | 59 | Possible | Carbo+Pem | Vin | 7 | 9 |
| 17 | Biphasic | M | 67 | Possible | Carbo+Pem | Trabectedin | 15 | 22 |
| 18 | Biphasic | M | 60 | Yes | Carbo+Pem | No | 6 | 8 |
| 19 | Sarcomatous | F | 80 | Yes | Carbo+Pem | Trabectedin | 3 | 5 |
| 20 | Sarcomatous | F | 78 | Unknown | Pem | No | 4 | 6 |
| 21 | Sarcomatous | M | 74 | Unknown | Carbo+Pem | No | 5 | 7 |
| 22 | Sarcomatous | M | 69 | Yes | Carbo+Pem | Trabectedin | 7 | 10 |
| 23 | Sarcomatous | M | 78 | Yes | Carbo+Pem | Trabectedin | 4 | 9 |

^a^ UPN: unknown patient number; M: male; F: female; Carbo: carboplatin; Pem: pemetrexed; CisPt:

**Additional Table 2. Histopathological features of the patients**

| **UPN** | **Histotype** | **CALRET** | **PANCK** | **Podo** | **EMA** | **CEA** | **WT1** | **CK5** | **BAP1** |
| --- | --- | --- | --- | --- | --- | --- | --- | --- | --- |
| 1 | Epithelioid | POS | POS | NEG | POS | NEG | POS | NEG | POS |
| 2 | Epithelioid | POS | POS | NEG | NEG | NEG | NEG | NEG | POS |
| 3 | Epithelioid | POS | POS | NEG | NEG | NEG | POS | POS | NEG |
| 4 | Epithelioid | POS | POS | NEG | NEG | NEG | NEG | NEG | NEG |
| 5 | Epithelioid | POS | POS | NEG | NEG | NEG | POS | NEG | POS N |
| 6 | Epithelioid | POS 50% | NEG | NEG | NEG | NEG | POS | NEG | POS N |
| 7 | Epithelioid | NEG | POS | NEG | NEG | NEG | POS | NEG | POS N |
| 8 | Epithelioid | POS | POS | NEG | NEG | NEG | NEG | NEG | POS N |
| 9 | Epithelioid | POS | POS | NEG | NEG | NEG | NEG | POS | POS N |
| 10 | Epithelioid | POS | POS | NEG | NEG | NEG | POS | POS | POS N |
| 11 | Epithelioid | POS | POS | NEG | spor POS | NEG | POS | NEG | NEG |
| 12 | Epithelioid | POS | POS | NEG | POS | NEG | POS | NEG | NEG |
| 13 | Epithelioid | POS | POS | NEG | NEG | NEG | POS | POS | NEG |
| 14 | Biphasic | POS | POS | NEG | NEG | NEG | POS | NEG | POS N |
| 15 | Biphasic | POS | POS | NEG | NEG | NEG | NEG | NEG | NEG 95% |
| 16 | Biphasic | POS | POS | NEG | NEG | NEG | POS | POS | NEG |
| 17 | Biphasic | POS | POS | NEG | NEG | NEG | FOC | NEG | POS N |
| 18 | Biphasic | POS | POS | NEG | NEG | NEG | POS | NEG | POS N |
| 19 | Sarcomatous | NEG | POS | NEG | NEG | NEG | NEG | NEG | NEG 95% |
| 20 | Sarcomatous | NEG | NEG | NEG | NEG | NEG | POS | NEG | POS N |
| 21 | Sarcomatous | FOC | FOC | NEG | NEG | NEG | FOC | NEG | NEG |
| 22 | Sarcomatous | NEG | POS | NEG | NEG | NEG | NEG | NEG | POS N |
| 23 | Sarcomatous | POS | POS | NEG | NEG | NEG | FOC | NEG | NEG |

^a^ UPN: unknown patient number; CALRET: calretinin; PANCK: pancytokeratin; Podo: podoplanin; EMA: epithelial membrane antigen; CEA: carcino-embryonic antigen; WT1: Wilms tumor-1 antigen; CK5: cytokeratin 5; POS: positive; NEG: negative; spor: sporadic; N: nuclear.

**Additional Table 3. Hematological parameters of the animals**

|  | **CTRL** | **MLN** | **PT** | **MLN+PT** |
| --- | --- | --- | --- | --- |
| RBC (x10^6^/µl) | 3.10±0.358 | 3.39±0.88 | 3.01±0.45 | 2.92±0.21 |
| Hb (g/dl) | 13.39±2.85 | 13.04±3.12 | 12.98±2.39 | 13.21±3.19 |
| WBC (x10^3^/µl) | 11.82±3.18 | 12.93±3.84 | 11.72±3.27 | 13.02±2.31 |
| PLT (x10^3^/µl) | 983±395 | 1092±391 | 938±182 | 1033±309 |
| LDH (U/l) | 6548±1028 | 5836±873 | 5983±562 | 6098±657 |
| AST (U/l) | 129±42 | 104±37 | 147±52 | 109±43 |
| ALT (U/l) | 47±10 | 43±11 | 42±17 | 47±13 |
| AP (U/l) | 109±37 | 127±37 | 137±23 | 126±31 |
| Creatinine (mg/l) | 0.024±0.008 | 0.031±0.004 | 0.035±0.009 | 0.036±0.008 |
| CPK (U/l) | 403±48 | 382±78 | 309±74 | 403±94 |

Balb/C mice (n=10/group) were treated as described in Figure 1. Blood was collected immediately after euthanasia and analyzed for red blood cells (RBC) counts, haemoglobin (Hb), white blood cells (WBC), platelets (PLT), lactate dehydrogenase (LDH), aspartate aminotransferase (AST), alanine aminotransferase (ALT), alkaline phosphatase (AP), creatinine, creatine phosphokinase (CPK). Data are means±SD. CTRL: control; MLN: MLN4924. PT: cisplatin.

**Additional Table 4. Genes mediating UPR modulated by MLN4924**

Primary sarcomatous BAP1^-^ (UPN22) MPM cells were incubated for 24 h in fresh medium (CTRL), with 50 μM cisplatin (PT), 0.2 μM MLN4924 (MLN) or their combination (PT+MLN) and analyzed for 84-relevant genes for UPR, in triplicates. The mean relative expression versus CTRL cells (considered 1) was calculated with the Gene Expression Quantitation software. p-value ≥0.05 was considered significant. Genes modulated > 2-fold are in bold characters. Grey cells: RT and PCR quality controls.

**Additional Table 5. Tumor mutation burden in mesothelioma cells treated with cisplatin, MLN4924 or combination**

| **Sample ID** | | **Exonic variants in target region** | **Target Region Size (Mb)** | **Tumor Mutational Burden (TMB)** |
| --- | --- | --- | --- | --- |
| **EPI** | **CTRL** | 462 | 49.475726 | 9.3379 |
|  | **PT** | 436 | 49.475726 | 8.8124 |
|  | **MLN** | 427 | 49.475726 | 8.6305 |
|  | **PT+MLN** | 424 | 49.475726 | 8.5699 |
| **BIP** | **CTRL** | 498 | 49.475726 | 9.5328 |
|  | **PT** | 471 | 49.475726 | 9.3276 |
|  | **MLN** | 441 | 49.475726 | 9.4452 |
|  | **PT+MLN** | 458 | 49.475726 | 9.2091 |
| **SAR** | **CTRL** | 551 | 49.475726 | 11.1368 |
|  | **PT** | 544 | 49.475726 | 10.9953 |
|  | **MLN** | 504 | 49.475726 | 10.9144 |
|  | **PT+MLN** | 537 | 49.475726 | 10.8538 |

Primary epithelioid (EPI BAP1^+^ UPN7), biphasic (BIP BAP1^-^ UPN16) and sarcomatous (SAR BAP1^-^ UPN22) MPM cells were incubated for 24 h in fresh medium (CTRL), with 50 μM cisplatin (PT), 0.2 μM MLN4924 (MLN) or their combination (PT+MLN). DNA was extracted and analyzed for the tumor mutational burden (TMB) by next generation sequencing. Each sample was analyzed in triplicates (n = 3).

**Additional Figure 1**

**
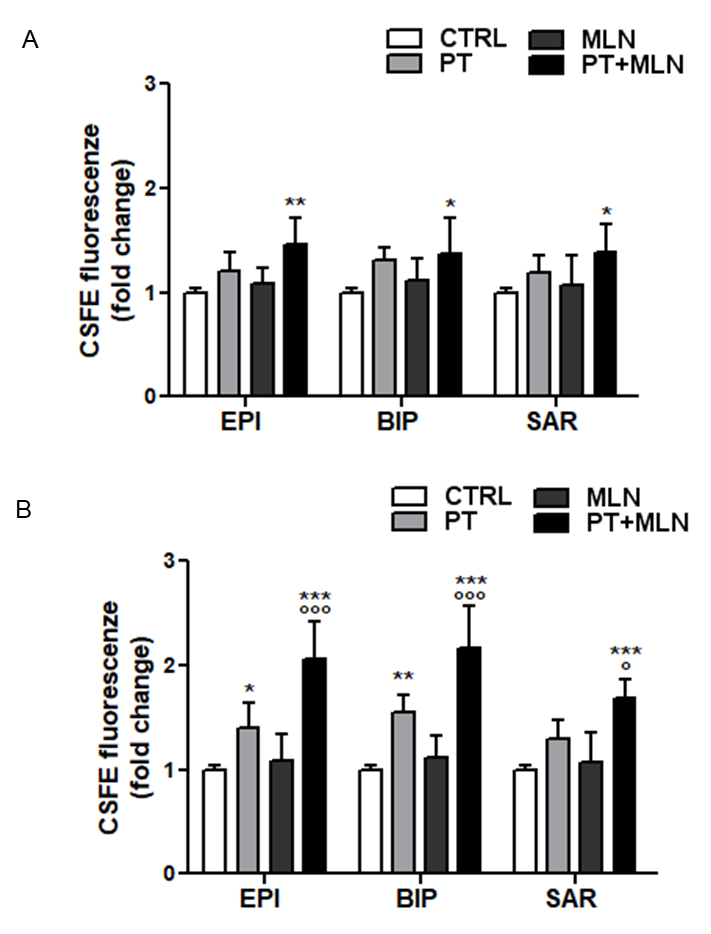
**

Additional Figure 1. Inhibition of proliferation induced by MLN+cisplatin combo. Primary MPM cells derived from 3 different histopathological subtypes, epithelioid (EPI BAP1^+^ UPN7), biphasic (BIP BAP1^-^ UPN16) and sarcomatous (SAR BAP1^+^UPN21) MPM, were incubated in fresh medium (CTRL), with 50 μM cisplatin (PT), 0.2 μM MLN4924 (MLN) or their combination (PT+MLN) for 24 h (A) or 48h (B). MPM cells proliferation was measured fluorometrically in triplicates using CSFE staining. Data are presented as means +SD (n = 3).

**Additional Figure 2**

**
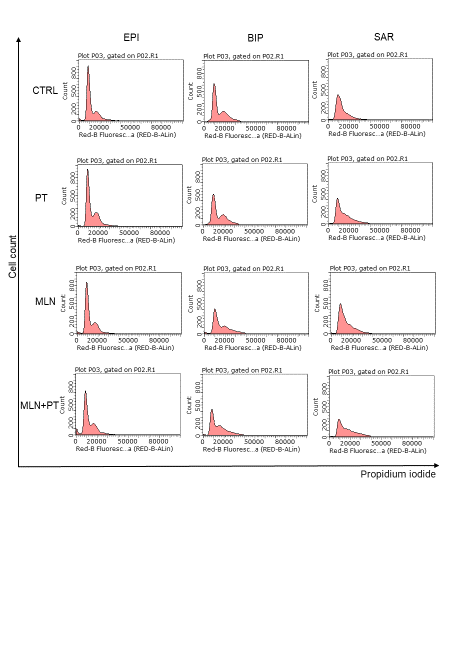
**

**Additional Figure 2. Representative histograms of cell cycle distribution**. Primary MPM cells derived from 3 different histopathological subtypes, epithelioid (EPI BAP1^+^ UPN7), biphasic (BIP BAP1^-^ UPN16) and sarcomatous (SAR BAP1^+^ UPN22) MPM, were incubated in fresh medium (CTRL), with 50 μM cisplatin (PT), 0.2 μM MLN4924 (MLN) or their combination (PT+MLN) for 24 h. Cell cycle distribution was measured by flow cytometry in duplicates.

**Additional Figure 3**

**
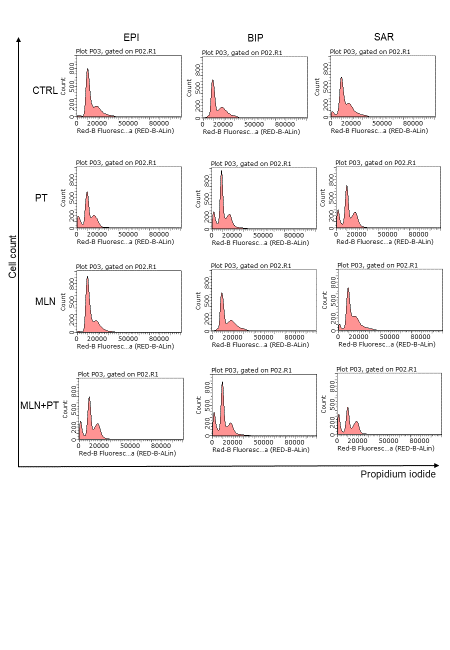
**

**Additional Figure 3. Representative histograms of cell cycle distribution**. Primary MPM cells derived from 3 different histopathological subtypes, epithelioid (EPI BAP1^+^ UPN7), biphasic (BIP BAP1^-^ UPN16) and sarcomatous (SAR BAP1^+^ UPN22) MPM were incubated in fresh medium (CTRL), with 50 μM cisplatin (PT), 0.2 μM MLN4924 (MLN) or their combination (PT+MLN) for 48 h. Cell cycle distribution was measured by flow cytometry in duplicates.

**Additional Figure 4
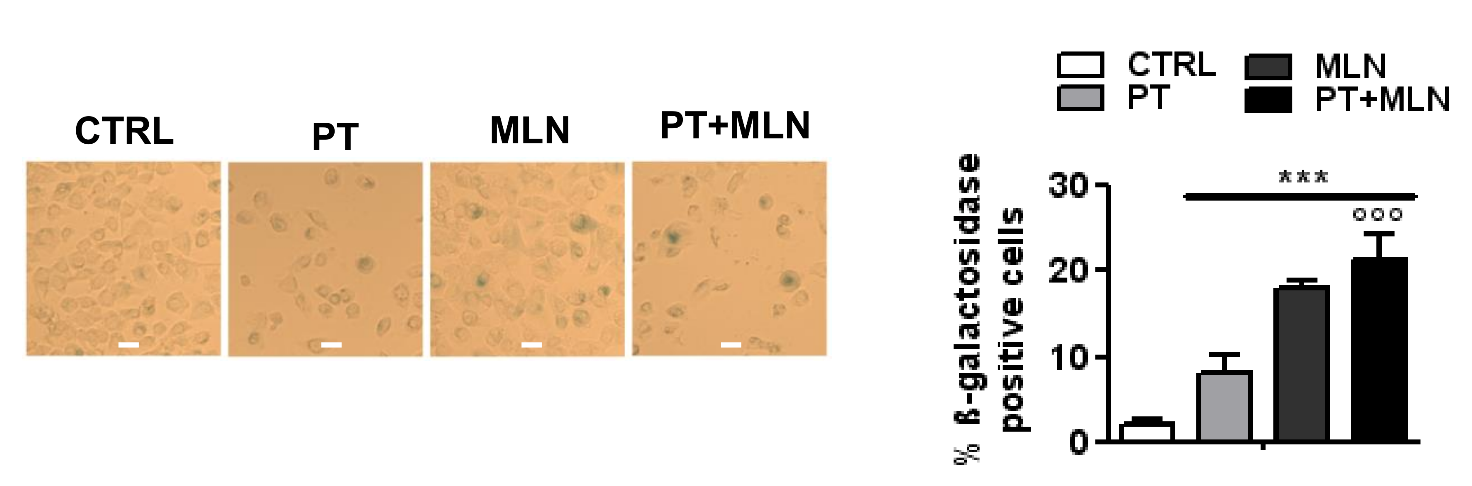
**

**Additional Figure 4 Senescence induced by MLN4924 in sarcomatous MPM cells.**

Primary sarcomatous (SAR BAP1^+^UPN21 and SAR BAP1^-^ UPN22) MPM cells were incubated in fresh medium (CTRL), with 50 μM cisplatin (PT), 0.2 μM MLN4924 (MLN) or their combination (PT+MLN). *Left panel*: representative photographs of senescent cells from UPN 22 detected by measuring β-galactosidase activity after 48 h of treatment. Scale bar 10 µm (10x ocular, 63x objective). At least 10 fields were examined for each condition. *Right panel*: quantification of β-galactosidase, performed with the ImageJ software. Data are presented as means + SD (*n* = 3). ****p* < 0.001: treated cells vs CTRL cells; °°°*p* < 0.01: PT+MLN-treated cells vs PT-treated cells. °

**Additional Figure 5**


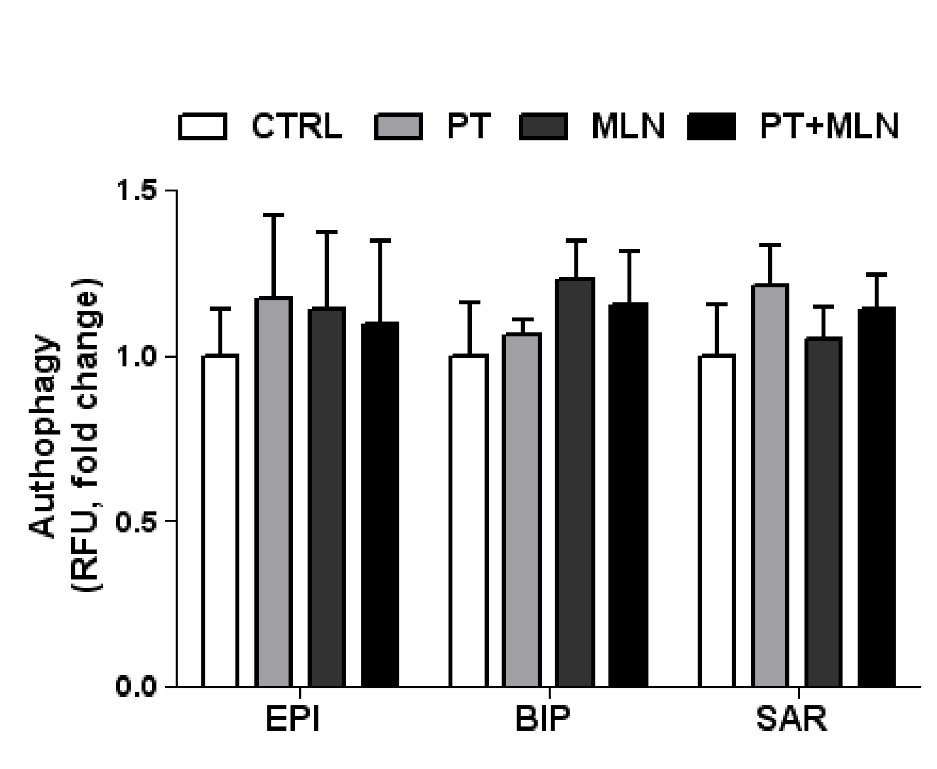


**Additional Figure 5. Effects of cisplatin and MLN4924 on autophagy**

Primary MPM cells derived from 3 different histopathological subtypes, epithelioid (EPI BAP1^+^ UPN7 and EPI BAP1^-^ UPN11), biphasic (BIP BAP1^+^ UPN14 and BIP BAP1^-^ UPN16) and sarcomatous (SAR BAP1^+^UPN21 and SAR BAP1- UPN22), were incubated in fresh medium (CTRL), with 50 μM cisplatin (PT), 0.2 μM MLN4924 (MLN) or their combination (PT+MLN) for 24 h. Autophagic flux was measured fluorometrically in triplicates. Data are presented as means from of 2 primary MPM for each histopathological subtype used + SD (n = 3).

**Additional Figure 6**

**
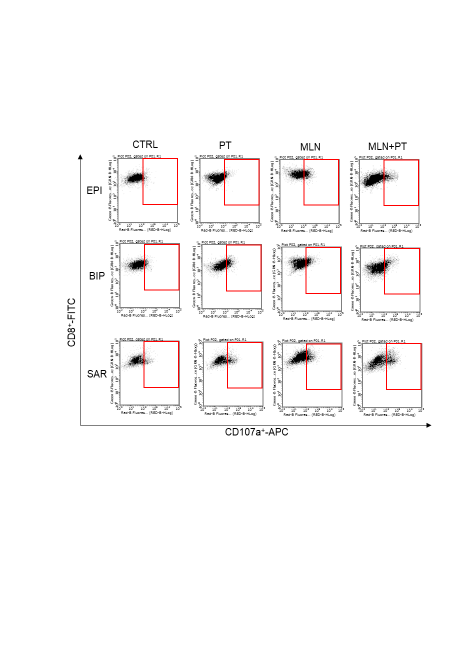
**

**Additional Figure 6. Representative dot plots of CD8^+^ 107a^+^ T-lymphocytes.**

T-lymphocytes, collected after co-culture with dendritic cells that have phagocytized MPM cells (incubated previously in fresh medium (CTRL), with 50 μM cisplatin (PT), 0.2 μM MLN4924 (MLN) or their combination (PT+MLN) for 24 h) were collected and stained for CD8 and CD107a, then analyzed by flow cytometry in duplicates. The figures represent the results from dendritic cells that have phagocytized epithelioid (EPI BAP1^+^ UPN7), biphasic (BIP BAP1^-^ UPN16) and sarcomatous (SAR BAP1^+^ UPN22) MPM. Red square: gate identifying CD107a^+^cells within CD8^+^ T-lymphocytes.

**Additional Figure 7
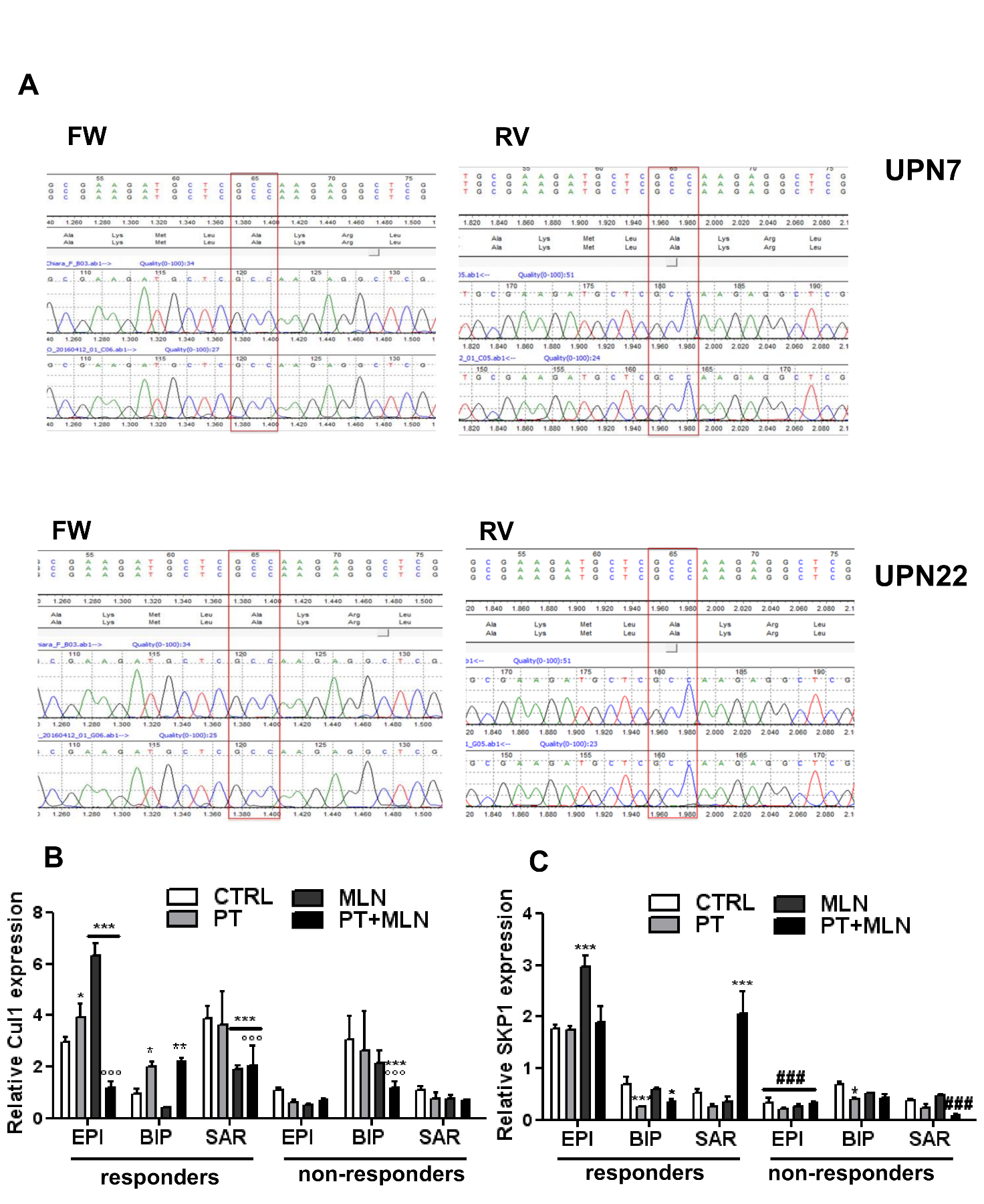
**

**Additional Figure 7. Cul1 mutational status and cullin1/SKP1 expression in MLN4924-responder and non-responder cells**

Top responder (the epithelioid EPI BAP1^+^ MPM UPN7, the biphasic BIP BAP1^-^ MPM UPN16, the sarcomatous SAR BAP1^+^ MPM UPN22) and top non-responder (the epithelioid EPI BAP1^+^ MPM UPN6, the biphasic BIP BAP1^+^ MPM UPN18, the sarcomatous SAR BAP1^-^MPM UPN21) cells were incubated 24 h in fresh medium (CTRL), with 50 μM cisplatin (PT), 0.2 μM MLN4924 (MLN) or their combination (PT+MLN). **A.** Sequencing of the *Cul1* hot spot mutation (codon 471) for the untreated top-responder UPN7 cells and top-non-responder UPN21. **B.** Cullin 1 mRNA levels were measured by RT-PCR, in triplicates. Data are presented as means + SD (*n* = 3). *p<0.05, **p<0.01, ****p* < 0.001: treated cells vs CTRL cells; °°°*p* < 0.001: PT+MLN-treated cells vs PT-treated cells. **C**. SKP1 mRNA levels were measured by RT-PCR, in triplicates. Data are presented as means ± SD (*n* = 3). *p<0.05, ****p* < 0.001: treated cells vs CTRL cells; °*p* < 0.05; °°°*p* < 0.001: treated cells vs PT-treated cells; ^###^*p* < 0.001: non-responder vs respective responder cells.
